# A systematic assessment of large language models’ knowledge of rare diseases: How much do large language models know about rare disease?

**DOI:** 10.1016/j.xhgg.2025.100558

**Published:** 2025-12-11

**Authors:** Tudor Groza, Allison J. Marcello, Tristan Carlisle, Weng Khong Lim, Melissa Haendel, Neerja Karnani, Peter N. Robinson, Holm Graessner, Jessica X. Chong, Gareth Baynam, Saumya Shekhar Jamuar

**Affiliations:** 1 Bioinformatics Institute (BII), Agency for Science, Technology and Research (A∗STAR), Singapore, Singapore; 2 Maternal and Child Health Research Institute, KK Women’s and Children’s Hospital, Singapore, Singapore; 3 Genetics Service, KK Women’s and Children’s Hospital, Singapore, Singapore; 4 SingHealth Duke-NUS Institute of Precision Medicine, Singapore, Singapore; 5 Division of Genetic Medicine, University of Washington, 1959 NE Pacific St, Box 357371, Seattle, WA 98195, USA; 6 Rare Care Centre, Perth Children’s Hospital, Perth, WA, Australia; 7 SingHealth Duke-NUS Genomic Medicine Centre, Singapore, Singapore; 8 Cancer & Stem Cell Biology Program, Duke-NUS Medical School, Singapore, Singapore; 9 Laboratory of Genome Variation Analytics, Genome Institute of Singapore, Agency for Science, Technology and Research (A∗STAR), Singapore, Singapore; 10 Department of Genetics, University of North Carolina at Chapel Hill, Chapel Hill, NC 27599-7264, USA; 11 Institute for Human Development and Potential, Agency for Science, Technology and Research (A∗STAR), Singapore, Singapore; 12 Department of Biochemistry, Yong Loo Lin School of Medicine, National University of Singapore, Singapore, Singapore; 13 Berlin Institute of Health, Berlin, Germany; 14 Institute for Medical Genetics and Applied Genomics, Centre for Rare Diseases, University Hospital Tübingen, Tübingen, Germany; 15 Brotman Bay Institute, Seattle, WA 98195, USA; 16 International Rare Diseases Research Consortium, Paris, France

**Keywords:** rare disease structured knowledge, Human Phenotype Ontology, rare disease HPO annotations, rare disease gene annotations, large language models

## Abstract

Large language models (LLMs) perform well on general medical benchmarks, but their ability to reason about rare diseases (RDs) remains unclear. Rather than challenge LLMs to diagnose a limited number of cases that are unlikely to represent all RDs or RD-associated genes, we instead sought to comprehensively probe LLM understanding of RD-associated genes and phenotypes. We systematically evaluated six leading general-domain LLMs (GPT-4, Claude 3.7, Llama-3.3 70B, Gemma-2 27B, Llama-3.2, and Phi-4) for their ability to generate core phenotypic features and causal genes required to support reasoning for 10,892 Orphanet diseases. Outputs were mapped to Human Phenotype Ontology (HPO) terms and HGNC gene symbols and compared with curated references using set overlap, semantic similarity, and disease ranking via the likelihood ratio interpretation of clinical abnormality (LIRICAL) framework applied to 8,000 patient Phenopackets. LLM recall of curated RD knowledge was generally low, with gene associations retrieved more accurately than phenotypes. Commercial models, particularly GPT-4 and Claude, achieved over 60% recall for gene associations but struggled with precise phenotype recovery. Despite low exact overlaps, moderate semantic similarity scores indicated partial alignment with curated data. When used in LIRICAL, LLM-derived phenotypic profiles yielded ranking performance close to that of gold standard profiles, although direct diagnostic accuracy remained limited. Interestingly, convergent non-curated terms across models suggest potential for hypothesis generation. Current generalist LLMs lack the precision to replace curated RD knowledge bases but offer complementary, semantically relevant information. Our results support hybrid approaches that combine expert curation with selectively integrated LLM outputs to enhance and scale ontology-driven RD diagnostics.

## Teaser

By probing LLM knowledge across 10,000 rare diseases, we show that models incompletely recall curated facts yet provide semantically aligned signals valuable for hypothesis generation and diagnostic support. This work clarifies LLM limitations and motivates hybrid approaches combining curation with selectively integrated model-derived knowledge.

## Introduction

Recent advances in large language models (LLMs) have produced encouraging results on well-established medical examinations,^1^^,^^2^^,^^3^^,^^4^ particularly multiple-choice vignette tests, yet the suitability of these systems for decision support in the rare disease (RD) domain remains largely uncertain. The RD knowledge landscape is semantically rich and accurate reasoning requires fine-grained integration of phenotype and genotype information. These differences raise the possibility that success on broad medical benchmarks may not translate to meaningful gains for the RD community.

Since the launch of OMIM^5^ in 1966 and GeneReviews in 1993, clinicians and researchers have relied on manually curated repositories to guide basic science and clinical care, from annotating gene function, selecting confirmatory tests, and interpreting genome-scale sequencing to guidelines for clinical care. The publication of the Human Phenotype Ontology (HPO) in 2008^6^^,^^7^ standardized the vocabulary for describing clinical features of RDs and, in combination with large curation efforts such as OMIM, the Monarch Initiative,^8^ and Orphanet,^9^ enabled algorithmic methods for patient matchmaking,^10^^,^^11^ variant prioritization,^12^^,^^13^ and disease ranking.^14^^,^^15^ In turn, these methods underpin most commercial diagnostic pipelines and large national sequencing programs, including Genomics England,^16^^,^^17^ and remain the reference point against which new computational approaches are judged. The RD curated knowledge is, however, the pillar upon which the entire ecosystem was built.

The growing adoption of LLMs in the last years prompts a natural question: what RD knowledge do LLMs possess, and can they help enrich the existing body of RD knowledge on which algorithms and tools depend on? Inspired by previous studies that examined LLM performance on diagnostic case reports^18^^,^^19^ and especially by RareBench—a real-world data-driven benchmark^20^—we performed a large-scale systematic assessment of the knowledge revealed by querying LLMs, under the assumption that this knowledge underpins their reasoning, explainability and disease-ranking capabilities. We attempted to gain an understanding in and quantify the degree of hallucinations produced in the form of phenotype and gene associations, but also the context in which such knowledge can indeed be used in combination with the existing high-quality manually curated resources.

We sampled six state-of-the-art general-domain LLMs (GPT-4, Claude 3.7, Llama-3.3 70 B, Gemma-2 27 B, Llama-3.2, and Phi-4) and asked them to propose core clinical features and causal genes for 10,892 Orphanet diseases (6,715 of which carry OMIM cross-references). The free-text outputs were mapped to HPO terms and HGNC gene symbols, enabling direct comparison with Orphanet, OMIM, and the HPO annotations (HPOA). Performance was quantified with exact set overlap, ontology-aware semantic similarity, and downstream disease-ranking impact, the latter assessed by substituting model-generated phenotypic profiles into the likelihood ratio interpretation of clinical abnormalities (LIRICALs) ranking framework^15^ across a cohort of 8,000 Phenopackets denoting patient profiles.^21^ It is worth noting that our focus was on revealing the knowledge encapsulated within the models combined with assessing the utility of this knowledge, rather than on their ability to perform certain tasks—such as phenotype concept recognition or disease ranking. Where previous studies^19^^,^^20^ evaluate how well LLMs perform RD diagnosis tasks using structured benchmarks and prompting strategies on patient data, our study complements this by systematically probing the raw, latent knowledge of generalist LLMs about RDs through free-text generation. By mapping these outputs to standard ontologies and testing their impact on downstream tasks, we reveal how LLMs’ internal knowledge can directly enrich existing community resources.

## Material and methods

**Figure 1:**
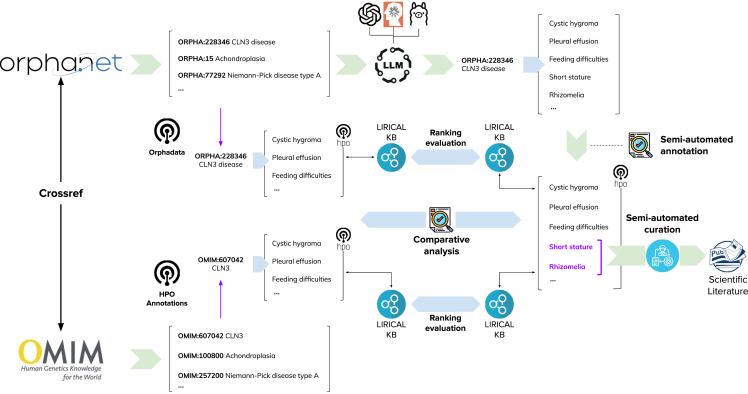
High-level overview of the methodology with application to phenotypes Six LLMs were prompted to externalize their knowledge on each of the 10,892 disease entries defined by Orphanet using the disease labels and synonyms (where available). The output was normalized to HPO concepts and compared against the disease-phenotype associations from Orphanet and HPOA. Evidence supporting novel disease-phenotype associations was compiled from PubMed abstracts. Finally, the annotation set produced by each model was used as a knowledge base underpinning the ranking mechanism provided by the LIRICAL framework and tested on a gold standard corpus of Phenopackets. The process was equivalent for genes, except for the LIRICAL ranking, which was omitted and the normalization process, which was performed manually using HGNC IDs.

Figure 1 depicts a high-level overview of our methodology with a focus on phenotypes. The process for genes was almost equivalent, except for the disease-ranking step involving the LIRICAL framework. Below we list the data and models underpinning this process and provide details for each step.

### Data and models

Our experiments were conducted using the HPO v.2025-03-03 for phenotype representation, the HPOA dataset (v.2025-03-03) and the Orphanet^9^ phenotype annotations (v.2024-12-03), respectively. High-level statistics on the two annotation datasets are listed in Table 1. Note that HPOA covers annotations for both OMIM^5^ and Orphanet identifiers. Our experiments used exclusively the annotations assigned to OMIM identifiers and relied on the original Orphanet annotations for the Orphanet experiments. Also, experiments analyzing gene annotations focused exclusively on causative genes and/or genes with documented pathogenic variants.

**Table 1:** High-level stats on the two datasets used as baseline for comparing the RD knowledge of the LLMs

Dataset	Disease identifiers (CURIEs)	Total no. of diseases	No. of diseases with phenotype annotations	Phenotypes annotation stats per disease	No. of diseases with causative gene annotations
Orphanet	ORPHA	10,892	4,281	mean: 19.65 median: 15 min: 0 max: 186	6,855
HPO annotations	OMIM	8,669	8,363	mean: 17.99 median: 13 min: 0 max: 211	7,472

The disease-ranking experiments relied on the LIRICAL framework^15^ v.2.0.4 (February 2025) and the Phenopackets corpus^21^^,^^22^ v.0.1.24 consisting of 8,174 clinical cases across 516 diseases. A Phenopacket denoting a clinical case had on average 20.53 HPO terms (median: 14; min: 1; max: 192). A disease part of the corpus had on average 15.84 patients (median: 5; min: 1; max: 462).

The LLMs evaluated by our method were

• OpenAI Gpt-4.o: Commercial; unknown number of parameters
• Anthropic Claude Sonnet 3.7: Commercial; unknown number of parameters
• Meta Llama-3.2: Free, 3B parameters
• Microsoft Phi4: Free, 14B parameters
• Google Gemma-2: Free, 27B parameters
• Meta Llama-3.3: Free; 70B parameters

The interaction with the commercial models was programmatic via their corresponding application programming interfaces (APIs). Llama-3.3 was interrogated using the Together.ai API. Experiments using the “small” LLMs (3B, 14B, and 27B parameters) were executed programmatically on a standalone MacBook Pro M2 Max (64GB RAM) using ollama v.0.6.8. The prompt used to collect the RD knowledge is provided under the LLM knowledge externalization prompt in the supplemental information and was executed individually for each of the 10,892 diseases defined by Orphanet.

### Alignment to HPO and phenotype evaluation

The evaluation of the quality of the phenotype produced by the models was preceded by an ontology alignment step and consisted of three components: comparative analysis, documentation of novel phenotypes, and evaluation in a ranking context.

#### Ontology alignment

To support the full validation workflow, all phenotype lists generated by the LLMs were aligned to standardized HPO concepts. This alignment was conducted iteratively to minimize the false positive rate. Initially, the textual phenotypes were processed using the FastHPOCR Python library (v.0.1.4),^23^ which produced a set of candidate HPO terms, their associated text snippets, and a list of residual (unmatched) phenotypes.

Each HPO term identified by FastHPOCR was validated using a voting mechanism. Specifically, the three small LLMs in our experimental setup (3B, 14B, and 27B parameter models) were prompted to assess whether the alignment between a textual phenotype and its matched HPO concept (including its label and synonyms) was accurate. Alignments that received unanimous agreement (three out of three votes) were retained; all others were discarded.

Residual phenotypes were subjected to an enrichment step before reprocessing. This involved prompting the three small LLMs to generate synonyms for each residual phenotype. The union of these synonyms was then re-analyzed using FastHPOCR to identify additional candidate HPO terms, which were again validated using the same voting process. This step also served to resolve potential conflicts arising from ambiguous synonym mappings to multiple HPO terms.

Phenotypes for which no valid HPO mappings could be identified were retained in the final dataset in their original textual form. To estimate the precision of our alignment pipeline, we manually reviewed the HPOA for a random sample of 500 diseases (∼5% of the disease corpus) and observed a false positive rate of 2.5%, which is substantially lower than current state-of-the-art benchmarks for this task.

As mentioned previously, we did not ask LLMs to perform a phenotype concept recognition task, but rather use the textual knowledge produced by them and aligned it using an existing library. There are several similar methods published in the literature^24^^,^^25^^,^^26^; however, we chose FastHPOCR, because at the time of performing our experiments it represented the state-of-the-art.^23^

#### Comparative analysis

The comparative analysis against existing knowledge bases relied on two metrics: (1) the Jaccard index—defined as the set intersection divided by the set union of two lists of HPO terms; and (2) a normalized semantic similarity metric (based on the Resnik metric). The equations used to compute the semantic similarity metric are listed below:

$$IC\left(t\right)=-\ln\frac{TF\left(t\right)}{N}$$

where *IC(t)* is the information content for term *t*, *TF(t)* is the frequency of the term *t* in a given corpus—in our case the number of disease annotations present in the HPOA dataset—and *N* is the total number of diseases in the corpus.

Given two sets of HPO terms S_1_ and S_2_, where S_1_ = {t_1_, t_2_, … t_m_}

$$sim\left(S_{1}\to S_{2}\right)=\frac{1}{m}\sum_{t_{1}\in S_{1}}\max_{t_{2}\in S_{2}}IC\left(MICA\left(t_{1},t_{2}\right)\right)$$

where MICA denotes the most informative common ancestor of two terms. The final similarity is computed by introducing symmetry:

$$sim\left(S_{1},S_{2}\right)=0.5*sim\left(S_{1}\to S_{2}\right)+0.5*sim\left(S_{2}\to S_{1}\right)\text{.}$$

The normalized similarity is computed by dividing this last equation to the sum of the individual similarity of a phenotype list as an identity set (i.e., *sim*(S_1_, S_1_) and *sim*(S_2_, S_2_)).

Similar to the concept recognition component, the literature consists of multiple options for semantic similarity metrics.^27^ The choice of using this symmetric Resnik variation was motivated by the fact that it is present in virtually all RD gene or disease prioritization approaches either directly as a component or as a baseline.

Statistical significance of the Jaccard index was computed using a hypergeometric test with a population size equal to the number of phenotype concepts in HPO and testing the assumption that the overlap is greater than expected.

#### Novelty assessment

To assess the validity of “novel” phenotypes identified by the LLMs, we leveraged the full corpus of scientific abstracts available on PubMed. A given HPO term was considered potentially novel if neither the term nor any of its parent or grandparent terms in the HPO hierarchy appeared in the reference set of disease annotations. For each disease associated with at least one such potentially novel phenotype, we queried PubMed to retrieve all relevant abstracts.

We then applied a pairwise validation procedure using the voting mechanism previously introduced for ontology alignment. Specifically, for each <abstract, disease, phenotype> triple, we prompted three small LLMs to assess whether the phenotype was described as a manifestation of the disease in the abstract (see the LLM phenotype relevance assessment prompt in the supplemental information). An association was deemed positively validated if all three models concurred in their judgment.

To enhance robustness, we conducted two additional manual checks.

• We manually reviewed all candidate associations where two of the three models voted positively, to identify potential false negatives.
• We manually validated a random sample of 400 (∼5%) from the total 8,067 pairs of novel disease-phenotype associations.

#### Ranking evaluation

The two methods discussed above offer a quantitative perspective for gap analysis between the knowledge externalized by LLMs and that curated over more than a decade by the RD community. To assess the quality of this knowledge, one can leverage disease-phenotype associations within an established disease ranking (or gene prioritization) framework. Although the literature is rich with such frameworks,^28^^,^^29^^,^^30^^,^^31^^,^^32^^,^^33^^,^^34^ very few are sufficiently flexible to allow integration of alternative knowledge bases.

Our aim was not to propose a novel ranking method, but rather to determine whether treating the LLM-derived output as a knowledge base within such a framework could yield disease-ranking (or gene prioritization) results comparable with those obtained using manually curated data. The only framework that permitted this experimental configuration was LIRICAL,^15^ which applies a likelihood ratio approach to compute pairwise phenotype-disease scores. These scores quantify the contribution of each phenotype to the ranking of a disease and are then used to calculate the probability of each disease in the knowledge base, given a patient’s phenotype profile.

To evaluate the quality of the phenotypes generated by the LLMs, we replaced the default LIRICAL knowledge base with LLM-generated knowledge bases—one at a time—and ran evaluations using the previously described Phenopackets corpus. Two subsets of the test corpus were used.

• A model-specific subset, containing only diseases for which phenotype associations were present in the corresponding LLM-derived knowledge base. This ensured that no test case required diagnoses unsupported by the input knowledge.
• A common subset, consisting of diseases represented across all knowledge bases, providing a consistent basis for direct comparison.

For each Phenopacket, LIRICAL was executed via its command-line interface using each knowledge base to generate a ranked list of candidate diseases. The top 10 ranked diseases were retained for analysis, with comparative results. Direct comparisons were made possible by the consistent use of Orphanet or OMIM codes across all knowledge bases.

### Gene evaluation

The gene evaluation process followed a similar trajectory to that of disease evaluation, with a few key differences.

• LLM-generated gene outputs were manually mapped and validated against HUGO Gene Nomenclature Committee (HGNC) identifiers.
• Due to the typically one-to-one nature of disease-gene associations, standard precision and recall metrics were used for comparative analysis, rather than the Jaccard index.
• Ranking evaluation was not performed for genes.

The novelty assessment employed the same methodology and prompt structure as in the disease evaluation, adapted appropriately for genes.

### LLM—Direct disease ranking

Various arguments can be made both for and against our assumption regarding the evaluation of phenotype quality generated by LLMs. The disease-ranking output is inherently dependent on the underlying method, and different methodologies may produce divergent rankings. Most existing approaches, such as those employing strategies similar to LIRICAL, which use HPO terms to represent phenotypes and rely on a disease-phenotype association knowledge base, also adopt comparable strategies to assess semantic similarity or proximity between the HPO terms in a patient’s phenotype profile and those within the knowledge base.^28^^,^^29^^,^^30^^,^^31^^,^^32^^,^^33^^,^^34^ We therefore argue that leveraging externalized phenotype knowledge through such methods enables a more accurate assessment of phenotype quality.

The architectures of the LLMs used in our experiments are based on fundamentally different principles from LIRICAL. However, their usage patterns in diagnostic or differential diagnosis tasks are similar: users input a list of phenotypes and expect a ranked list of candidate diseases. To allow a direct comparison with LIRICAL, we prompted six LLMs to generate a ranked list of conditions based on phenotype profiles from the Phenopackets gold standard corpus (see the LLM direct diagnosis prompt in the supplemental information). From an experimental standpoint, the input and expected output were identical; the key difference lies in the method used to generate the response. It is critical to note that we performed this task solely to provide a comparative ground; our main aim was not to assess the efficiency of the LLMs to diagnose RDs.^19^^,^^20^

A major challenge in comparing the LLM-generated ranked lists to the gold standard was their inability to produce disease names according to a standardized disease nomenclature. To address this, we manually mapped all disease names in the LLM outputs to Orphanet terms. Evaluation was then conducted at two levels.

• Exact matching—where the Orphanet disease ID in the LLM output matched exactly the Orphanet (or OMIM-equivalent) ID in the gold standard.
• Broader matching—where the Orphanet disease ID in the LLM output corresponded to a broader disease category, and the gold standard disease was a subtype. For instance, an LLM may have returned “*Developmental and epileptic encephalopathy*,” whereas the reference diagnosis was “*Developmental and epileptic encephalopathy 4*” or “*Developmental and epileptic encephalopathy 11.*”

## Results

### LLMs do not reproduce the phenotype/genotype knowledge currently documented by manually curated resources

**Figure 2:**
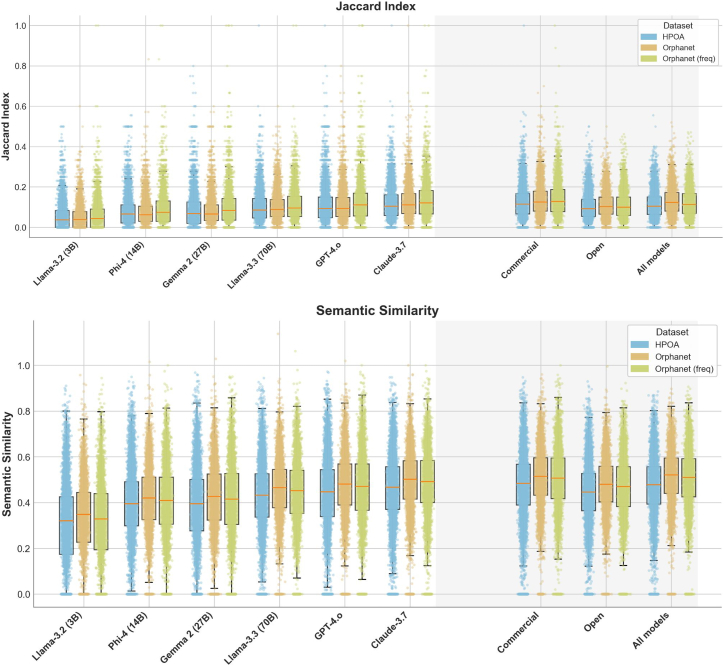
Distributions of pairwise Jaccard index and semantic similarity between the phenotype sets produced by various LLMs and reference knowledge bases HPOA (HPO annotations), Orphanet and Orphanet (freq)—the latter denoting the subset of Orphanet retaining only the frequent, very frequent, and obligate phenotypes

Across every system assessed, two commercial LLMs (GPT-4 and Claude 3.7) and four openly licensed models of varying scale, the overlap between generated phenotype sets and the reference annotations in HPOA/OMIM or Orphanet was modest at best. Using a strict set-based comparison, median Jaccard indices were below 0.10 for all six models (Figure 2), falling to <0.05 for three of the smaller open-weights models. Restricting the benchmark to Orphanet phenotypes labeled frequent, a subset that should in principle be better represented in information published on the web, did not materially improve performance (median 0.11 for Claude 3.7, 0.09 for GPT-4). These observations confirm that even extensive web crawl pre-training fails to reproduce the phenotype inventories that clinicians rely upon for day-to-day diagnostic reasoning. The concrete values supporting Figure 2 are listed in Table S1.

A term-aware comparison based on Resnik semantic similarity softened, but did not eliminate, this discrepancy. Commercial models reached median similarities of ≈0.55 across HPOA, Orphanet, and the combined datasets, whereas open models clustered around 0.40. The similarity distribution, however, exhibited a steep right-truncation: raising the acceptance threshold from 0.40 to 0.60 reduced coverage by more than half for all systems (Figure S1). Moreover, alignment was systematically higher for diseases present in both Monarch/HPOA and Orphanet than for Orphanet-unique entries, indicating that gaps in the training data disproportionately affect conditions curated by the European resource.

Organ-system coverage was uneven. An analysis of organ-system representation revealed additional heterogeneity. At a semantic similarity cut-off of 0.55 (Figure S2) auditory, ophthalmic, neurological, and developmental phenotypes accounted for the bulk of mismatches, with several diseases in these categories receiving zero high-confidence terms from three or more models. Inspection of term frequency distributions showed that commercial models tended to generate broader phenotype sets, whereas smaller open models produced shorter lists skewed toward highly general terms such as “Intellectual disability” or “Global developmental delay.” These tendencies reduced Jaccard overlap but improved semantic similarity and may partly explain the models’ performance in downstream tasks. Concrete examples of well-aligned diseases include: Li-Fraumeni syndrome, familial short QT syndrome, or catecholaminergic polymorphic ventricular tachycardia (additional examples are listed in Table S2).

**Figure 3:**
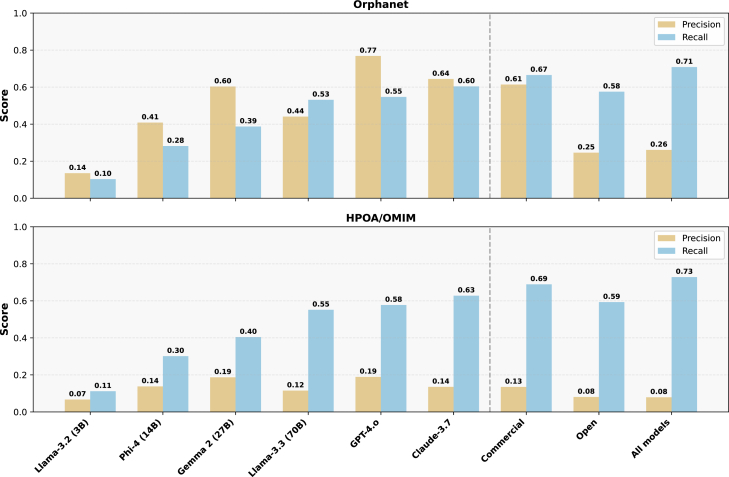
Precision and recall achieved by the various LLMs on reproducing the causative genes associated with the diseases documented by the reference knowledge bases Orphanet and HPOA

Gene-disease links were recovered more faithfully than phenotypes. In contrast to the heterogeneous phenotype results, canonical gene-disease links were recovered with considerably higher fidelity. Claude 3.7 attained a median recall of 0.64 and GPT-4 0.61 against the union of OMIM and Orphanet gene sets (Figure 3). The open models spanned 0.35–0.50, largely reflecting parameter count. Notably, precision lagged behind recall for every system: each model contributed a non-trivial set of novel genes per disease. Because gene lists lack hierarchical structure, this pattern implies genuine over-prediction rather than taxonomic substitution.

### There is signal in the noise

**Figure 4:**
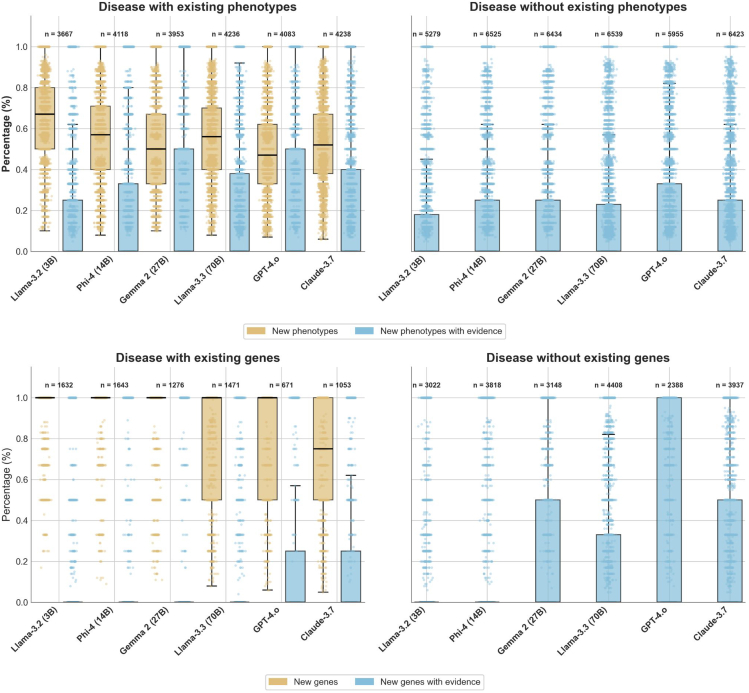
Distributions of the percentage of new phenotypes and new phenotypes with scientific evidence and new genes and new genes with scientific evidence across diseases already associated with phenotype/genes and diseases without annotations

To quantify the credibility of these additional outputs we searched PubMed for evidence supporting each new phenotype or gene (see novelty assessment in the materials and methods). While the median percentage of evidence was 0 across all models for both phenotypes and genes, the commercial models produced a longer tail of HPO terms and genes that had literature support (Figure 4). This aspect was particularly visible in conditions that had no annotations or genes recorded in HPOA or Orphanet. The concrete values supporting Figure 4 are listed in Table S3.

Note that, in the case of diseases without curated phenotypes or genes, the models tend to generate a greater number of disease-gene associations with potential evidence than disease-phenotype associations. Gene-disease relationships are more frequently discussed in molecular and mechanistic research literature, allowing LLMs to infer potential links from mentions of gene function, pathways, or experimental results even when phenotypic data are missing. In contrast, disease-phenotype associations are typically described in clinical narratives, case studies, or cohort reports, which are often scarce or behind paywalls for many RDs. This limited availability of patient-level clinical evidence constrains the model’s ability to generalize or infer phenotype associations, whereas gene-level relationships can still be derived from broader molecular context and cross-disease analogies.

Despite the low absolute hit rate, convergence analysis uncovered a distinct subset of shared suggestions: gene or phenotype additions proposed independently by at least two commercial models but absent from both curated databases and the abstract-level literature. Median Jaccard overlap on these novelties was 0.23 for phenotypes and 0.19 for genes (Figures S3–S6). While many overlaps undoubtedly represent correlated hallucinations driven by similar pre-training data, others may flag legitimate associations that have yet to reach the curation pipeline. This pattern will be discussed in more depth in the discussion.

### LLM knowledge is surprisingly complementary

**Figure 5:**
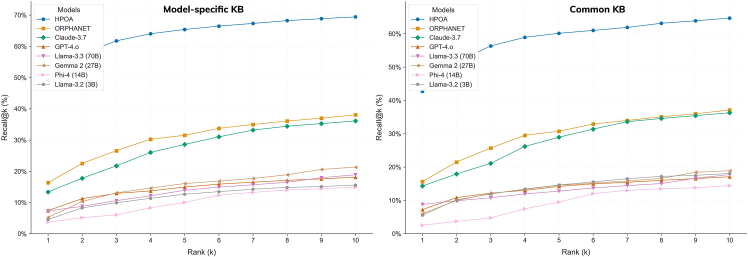
Evaluation of the phenotype sets produced by the LLMs on a diagnosis/differential task using a Phenopackets gold standard corpus Here, phenotype sets produced by the LLMs are used to create a knowledge base, which then supports disease ranking using the LIRICAL framework. The knowledge base was varied to include all diseases covered by the LLM from the gold standard vs. a subset that was common to all.

**Figure 6:**
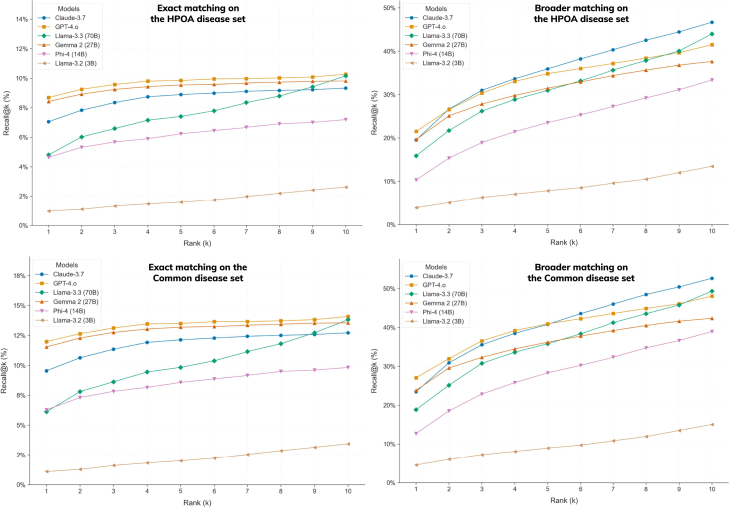
Evaluation of the phenotype sets produced by the LLMs on a diagnosis/differential task using a Phenopackets gold standard corpus via direct prompting of the patient profiles covered by the HPOA knowledge base and the Common set Two disease-matching strategies were employed to account for a positive hit: exact name matching and broader name matching (i.e., matching at the level of the disease group name).

We next asked whether noisy LLM annotations could nevertheless assist existing disease-ranking tools. The HPOA files used by the LIRICAL framework^15^ matcher were replaced with each model’s disease-phenotype knowledge sets, and disease ranking was evaluated on a corpus of 8,000+ patient Phenopackets covering 514 conditions^21^^,^^22^ (see Table S4). Compared with the HPOA baseline, recall at ranks 1, 5, and 10 fell for all model knowledge sets, but the knowledge derived from the best-performing system (Claude) matched the recall achieved when LIRICAL used Orphanet annotations, and GPT-4 was close (Figure 5). Notably, when the same LLMs were asked directly to propose a differential diagnosis for the identical Phenopackets, performance fell sharply; recall at 10 rarely exceeded 10% (Figure 6). The discrepancy suggests that, even though individual models cannot accurately identify a diagnosis, their aggregate “knowledge” can still boost a phenotype-driven ranking mechanism that relies on ontology-based similarity.

Relaxing the matching criterion to accept broader disease categories—such as grouping developmental and epileptic encephalopathies into a single entry—improved recall across the board, and the disease coverage of LLM-backed rankings was roughly three times higher than that obtained through direct querying (as shown in Figures S7 and S8). Note that the Jaccard index and the semantic similarity metric values for the set of diseases underpinning the Phenopackets corpora followed the same pattern as the entire dataset—see Figures S9–S12. Together, these observations support a view of LLM output as complementary rather than redundant: it is not an accurate mirror of curated knowledge but contains enough semantically coherent information to add value when filtered through established ontologies and similarity metrics. Examples of the top 10 ranked conditions include Holt-Oram syndrome, Greig cephalopolysyndactyly syndrome, Pallister-Hall syndrome, or Aarskog-Scott syndrome (the entire listing is presented in Table S5).

## Discussion

In the following we dive deeper into two areas that could be regarded as limitations of our study: the level of coverage and granularity of our experimental setup and results and the utility of the knowledge externalized by the LLMs in the context of hallucinations and data leakage.

Our aim was to investigate the added value brought by LLMs in enriching RD knowledge in the entirety of the domain’s size and spread. Orphanet defines 10,892 rare conditions (as per the data export listed in the materials and methods), which can be grouped in various ways—for example by considering their genetic etiology (if this exists) or by taking into account the prevalence. We proceeded with performing this grouping to produce outcomes comparable with those depicted in Figure 2. In addition, we focused on genetic conditions (as per the Orphanet definition) and attempted to test the correlation between the prevalence of a disease and the similarity of the generated profile vs. the profile documented by Orphanet.

These additional analyses paint a much more encouraging picture than the Jaccard values depicted in Figure 2. When we stratified the LLM-generated annotations by broad disease type (genetic vs. neoplastic vs. other; Figure S13) and by prevalence (Figure S14), both the Jaccard- and the Resnik-based normalized semantic similarity displayed remarkably similar values to those in Figure 2. It is, hence, striking to note what the data does *not* show: no meaningful correlation between disease type or prevalence and either similarity metric. A closer look at the association between the disease classification and the similarity values (using Orphanet as base) showed that endocrine, neurological, and renal disorders benefited from LLM enrichment to almost exactly the same extent as dermatological or ophthalmic conditions, despite the very different amounts of legacy curation those areas have accumulated. The uniformity across all disease classes suggests that the transformer pre-training objective captures latent cross-system structure that manual pipelines, constrained by domain expertise silos, potentially miss. To exemplify, ultra-rare entities such as pseudopseudohypoparathyroidism (endocrine) or Leydig-cell hypoplasia (urogenital) achieved similarity scores that were on par with comparatively common disorders such as tuberous sclerosis complex (skin) or Waardenburg syndrome type 2 (developmental anomalies during embryogenesis). Similarly, galactosialidosis (ophthalmic/inborn errors of metabolism), myoclonus-dystonia syndrome (neurological), or C3 glomerulopathy (renal) matched the similarity values of Eales disease (ophthalmic) or idiopathic bronchiectasis (respiratory), the latter being more common. A complete listing is presented in Table S6. Together, these observations refute the intuitive but inaccurate assumption that LLMs merely mirror web frequency signals. Instead, they appear to generalize patterns in biomedical language in a way that treats prevalence-rich and prevalence-poor diseases more equitably.

This breadth has practical consequences for maintaining RD knowledge bases. Because coverage gains are *evenly distributed*, curators can ingest LLM-suggested phenotypes or gene links confident that the uplift will be felt across the catalog, not just in already well-served areas. Moreover, the long-tail of legitimate, literature-supported suggestions (Figure 4) emerges in virtually every disease category, reinforcing the idea that large models can surface niche insights that manual triage might otherwise overlook. The fact that two independent commercial systems converge on a non-trivial subset of these novelties further bolsters their plausibility and provides a simple consensus filter to prioritize curation effort.

The second area of concern is related to hallucinations and data leakage. Starting with the latter, data leakage—while problematic in a disease-ranking context due to the inability to discern whether the result produced by the model is genuinely “computed” or just retrieved from the training data—does not carry a negative connotation in our context. There are a variety of data sources available on the web—and, hence, subject to the RD resource being enriched, augmentation with knowledge “retrieved” by LLMs from such other sources leads to a positive outcome.

In terms of hallucinations, when a model proposes a phenotype-disease or gene-disease link absent from reference databases, the default assumption of falsehood may in fact be inappropriate: the RD literature is sparse, publication lag is real, and many legitimate associations exist only in case reports. On the other hand, manual curation is tedious, time consuming and subjected to the limited human resources available to perform it. At a high level, our analysis shows that commercial models converge on a subset of these putative novelties, suggesting that at least some of the “noise” carries authentic signal.

In practice, a pattern emerges from the data when combining outcomes produced by multiple LLMs, which can lead to a potential solution of integrating this knowledge into established resources: gene or phenotype associations proposed independently by at least two commercial models that are shared provide good candidates for curation (as discussed above and depicted in Figures S3–S6). To support this claim we performed two tasks. Under the assumption that phenotypes belonging to top-level HPO abnormalities (i.e., musculoskeletal, growth, digestive, respiratory, etc.) not covered by an existing knowledge base, such as Orphanet, would have a higher chance to represent real hallucinations, we sampled and manually reviewed 130 entries (10%) from a total of 1,300 entries generated by the 2 commercial LLMs in the context of the Orphanet genetic diseases. Figure S15 considers Orphanet as a reference point and shows that, with the exception of Llama-3.3, commercial models yield a higher number of such associations of previously not covered top-level HPO abnormalities. The manual review resulted in a 42.3%–30.7%–27.0% split between phenotypes with scientific evidence (i.e., published case reports), invalid associations, and associations already covered by OMIM (e.g., short stature in coxopodopatellar syndrome). As a result, a third of the generated phenotypes represented real hallucinations, although this is only partly correct, since in several cases such phenotypes were secondary to the disease, e.g., respiratory distress in COFS syndrome. Concrete new associations (not covered by Orphanet and OMIM) include:

• nephronophthisis in cranioectodermal dysplasia^35^
• failure to thrive in epidermolysis bullosa simplex with pyloric atresia^36^
• lower limb asymmetry in Maffucci syndrome^37^
• growth delay in mosaic trisomy 1 [https://doi.org/10.31901/24566330.2008/08.04.01]
• abnormal heart morphology in trisomy 5p^38^

Similar outcomes have been noted also for genes, such as

• *AIRE* in chronic mucocutaneous candidiasis^39^
• *TPM3* in severe congenital nemaline myopathy^40^
• *CEP290* in Joubert syndrome with ocular defect^41^
• *FOXG1* in atypical Rett syndrome^42^
• *EDNRB* in Hirschsprung disease^43^

**Figure 7:**
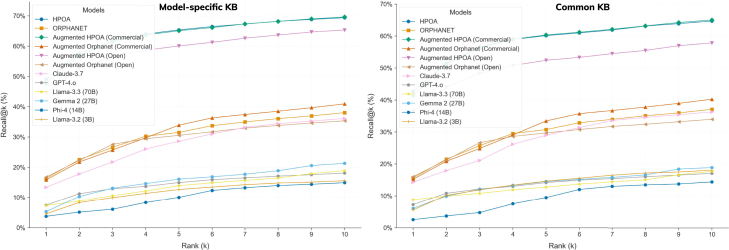
Evaluation of the phenotype sets produced by the LLMs on a diagnosis/differential task using a Phenopackets gold standard corpus repeated by augmenting the base models (HPOA and Orphanet) with phenotypes found in common across the commercial models and separately by the open models These results are directly comparable with those depicted in Figure 5 and show the added value of enriching the existing RD resources with LLM-generated knowledge.

A second experiment was to test the utility of the common phenotypes in the context of disease ranking. Consequently, we augmented the base HPOA and Orphanet models with new phenotypes found in common by the commercial models (Claude and GPT) and, separately, by the open models. The results depicted in Figure 7 show that, in both cases, although to a much smaller extent in the case of HPOA (∼0.5%), the augmented (commercial) KBs improved the results achieved by their corresponding base KBs.

A key takeaway from our work is that the definition of hallucinations is inherently task dependent. In the context of knowledge enrichment, what might traditionally be labeled as hallucinations may in fact represent reasonable and testable hypotheses. Importantly, our findings suggest that confidence in such hypotheses should increase proportionally with the number of LLMs that independently propose them, particularly when this includes a high-performing commercial model. This is complemented by our results arguing to view the output of LLMs as a uniform, *prevalence-agnostic enrichment layer*, which, leveraged judiciously, can accelerate the expansion of RD resources and narrow historical coverage gaps.

To responsibly integrate this type of knowledge into established resources, we propose introducing a new category within the curation process. This category would function as a form of “second-class citizen,” allowing potentially valuable but as-yet unvalidated information to be distinguished from fully verified entries. Users would then have the discretion to determine how best to utilize these provisional hypotheses. This approach parallels practices such as Orphanet’s inclusion of genes that are listed as potentially causative for RDs, despite the current lack of definitive evidence (i.e., “Candidate gene tested in” relations in the Orphanet-Genes KB).

Before concluding, we discuss some of the concrete limitations of our study. Our phenotypic analysis was confined to terms that could be mapped to the HPO. Unmappable strings were rare (see Figure S16—median of zero per disease) and thus unlikely to alter the main conclusions, but they may include valuable clinical nuances.

The ability of our automated pipeline to translate free-text phenotypes into valid HPO terms acts as a potential confounder. After normalization, a median of 95% of model-generated phrases mapped successfully to at least one HPO identifier (Figure S16). The remaining 5% comprised either synonyms not yet represented in HPO or multi-concept strings without straightforward decomposition. Notably, entries that did map often expanded into multiple HPO terms, yielding total phenotype counts exceeding 100% of the original line count. These expansions mimic the curation practices of Orphanet and HPOA and likely explain why model phenotype lists, although noisy, contain sufficient granular detail to drive similarity-based ranking.

It is also important to note that phenotypic and genotypic knowledge is most likely represented at markedly different levels of depth within LLMs. Genes, typically referenced through standardized HGNC symbols, are encountered more frequently and consistently in model training corpora, resulting in more stable and robust internal representations. In contrast, phenotypes are described with greater linguistic variability and contextual nuance. This diversity of expression introduces fragility in how LLMs capture and normalize phenotypic knowledge. Unlike genes, which exist as immutable, atomic identifiers, phenotypic concepts can be phrased in multiple synonymous or paraphrased forms, necessitating dedicated normalization approaches such as FastHPOCR. In summary, we expect LLMs to be able to recall RD genes more consistently than RD phenotypes, which is consistent with the results depicted in Figure 4.

Our literature validation relied on PubMed abstracts. Some claims that appeared unsupported may be documented in full-text articles, which may have been covered by the training data used by commercial models and hence raises the question of whether these should really be discarded as hallucinations. The performance of LLM-based systems may be improved by using techniques such as prompt engineering, retrieval augmented generation, fine-tuning, and agentic approaches. Therefore, our results may represent a lower bound to the performance of LLMs on the task of creating HPO-based computational disease models.

Taken together, the results indicate that, while contemporary LLMs are not a replacement for expert curation in RD genomics, they hold significant potential for safely enriching existing knowledge sources and bioinformatics tools. By integrating LLM-generated insights with structured resources such as HPO-based matchers, we can meaningfully expand and update the current knowledge landscape. On the other hand, knowledge concerning RDs is continually expanding, with newly reported cases contributing to the progressive enrichment of this domain. The performance of LLMs is inherently influenced by the extent to which their training corpora encompass such RD scenarios. Consequently, when analyzing diseases and clinical cases across different temporal periods, a time-stratified evaluation may reveal that LLMs generally exhibit lower performance on recently characterized or newly defined RDs, reflecting the lag between the emergence of novel medical knowledge and its incorporation into model training data.

Ensuring the safe and effective incorporation of these models will require purpose-built RD benchmarks that reflect real-world curation tasks, as well as evaluation frameworks that differentiate unsupported fabrications from low-evidence but potentially valuable hypotheses. Continued development of auditable mid-size models will further strengthen trust in this process. A hybrid strategy, where curated ontologies provide a robust foundation and LLMs contribute context-sensitive hypotheses and connections, offers a promising avenue for sustaining and enhancing the accuracy, depth, and utility of bioinformatics tools for the RD community.

Finally, given that LLMs are frequently utilized by individuals with RDs and their caregivers or advocates as tools for information seeking and hypothesis generation, it is important to acknowledge that they may propose novel phenotype-disease or gene-disease associations that are not yet represented in established reference databases. While such outputs should be interpreted with appropriate caution, the default assumption that these associations are false may be unwarranted, as they could reflect emerging or underreported medical knowledge. This consideration warrants explicit discussion when evaluating the potential and limitations of LLM-assisted reasoning in RD contexts.

## Data and code availability

The datasets generated during this study are available in GitHub at https://github.com/tudorgroza/code-for-papers/tree/main/llm-rdassess.

## Acknowledgments

S.S.J. is supported by National Medical Research Council Clinician Scientist Awards NMRC/CSAINVJun21-0003 and NMRC/CSAINV24jul-0001. T.G. is supported by NMRC/CSAINVJun21-0003 and by the 10.13039/501100001349National Medical Research Council Research, Innovation and Enterprise (RIE2025) Center Grant seed funding (NMRC/CG1/006/2021-KKH). W.K.L. is supported by National Precision Medicine Programme (NPM) Phase II funding (MOH-000588). J.X.C. and A.J.M. were supported by R35HG011297and J.X.C. was supported by U01HG011744.

## Author contributions

Conceptualization of research aims and goals, T.G., P.N.R., H.G., J.X.C., G.C., and S.S.J.; methodology development, T.G., A.J.M., T.C., P.N.R., and J.X.C.; validation and rigor, T.G., W.K.L., M.H., N.K., P.N.R., N.K., H.G., G.B., and S.S.J.; analysis and interpretation, T.G., M.H., P.N.R., and J.X.C.; investigation and collection of data, T.G., A.J.M., T.C., W.K.L., and J.X.C.; data curation, T.G., A.J.M., T.C., P.N.R., and J.X.C.; writing – original draft, T.G., J.X.C., N.K., P.N.R., G.B., and S.S.J.; funding acquisition, S.S.J., G.B., and N.K. All authors read and approved the final manuscript.

## Declaration of interests

J.X.C. serves as the Deputy Editor of *Human Genetics and Genomics Advances* and T.G. serves as a member of the Editorial Board of *Human Genetics and Genomics Advances*.

## Web resources

Online Inheritance in Man (OMIM), https://omim.org/
GeneReviews, https://www.ncbi.nlm.nih.gov/books/NBK1116/
HPO annotations, https://hpo.jax.org/
Orphanet data, https://www.orphadata.com/orphanet-scientific-knowledge/
Phenopackets gold standard, https://github.com/monarch-initiative/phenopacket-store

## Supplemental information

Document S1. Figures S1–16 and Tables S1–S6

Document S2. Article plus supplemental information
