# Supplementary material for: A systematic assessment of large language models’ knowledge of rare diseases: How much do large language models know about rare disease?
Source: HGG Adv. 2025 Dec 11;7(1):100558. doi: 10.1016/j.xhgg.2025.100558 (PMC12796007; doi:10.1016/j.xhgg.2025.100558)
Supplement: Document S1. Figures S1–16 and Tables S1–S6 [file mmc1.pdf]

HGGA, Volume 7

Supplemental information

A systematic assessment of large language models' knowledge of rare diseases: How much do large language models know about rare disease?

Tudor Groza, Allison J. Marcello, Tristan Carlisle, Weng Khong Lim, Melissa Haendel, Neerja Karnani, Peter N. Robinson, Holm Graessner, Jessica X. Chong, Gareth Baynam, and Saumya Shekhar Jamuar

Supplementary material

Fig. S1. Disease coverage based on the semantic similarity of the phenotype sets across models and datasets. A steep decline is shown with the gradual increase of the threshold in similarity. Commercial and combo (*All*, *Open*, *Commercial*) models exhibit a better performance. Combo models were created by performing a set union of the phenotypes across base models. *Commercial* includes Claude and GPT, *Open* the other 4 and *All* – all models.

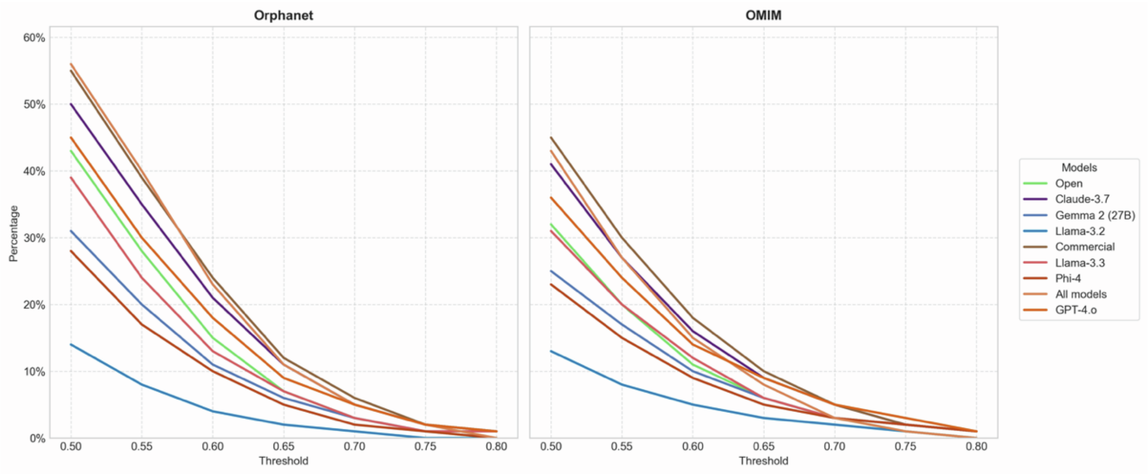

Fig. S2. Distribution of disease groups according to the Orphanet classification at a phenotype semantic similarity threshold of 0.55. The charts display a lack of correlation between the types of diseases underpinned by the models as a direct result of the phenotypes they externalize

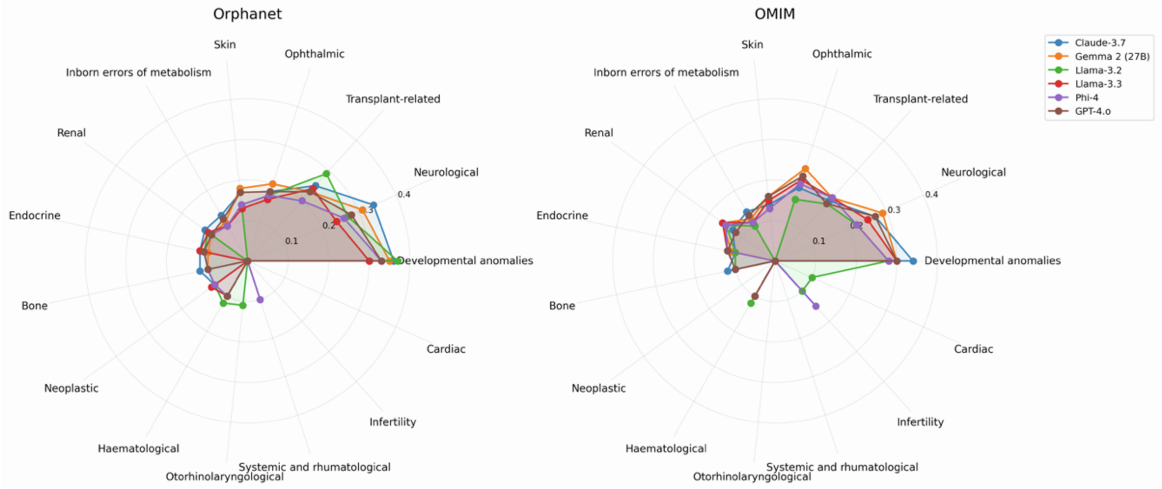

Fig. S3. Overlap analysis and comparison against the HPOA dataset with a focus on externalized "novel" phenotypes, when considering incrementally larger subsets of LLMs. Unsurprisingly, the median Jaccard index decreases with the number of models involved in the overlap analysis. Also, unsurprisingly, the agreement between Commercial models is markedly higher than the rest of the combinations.

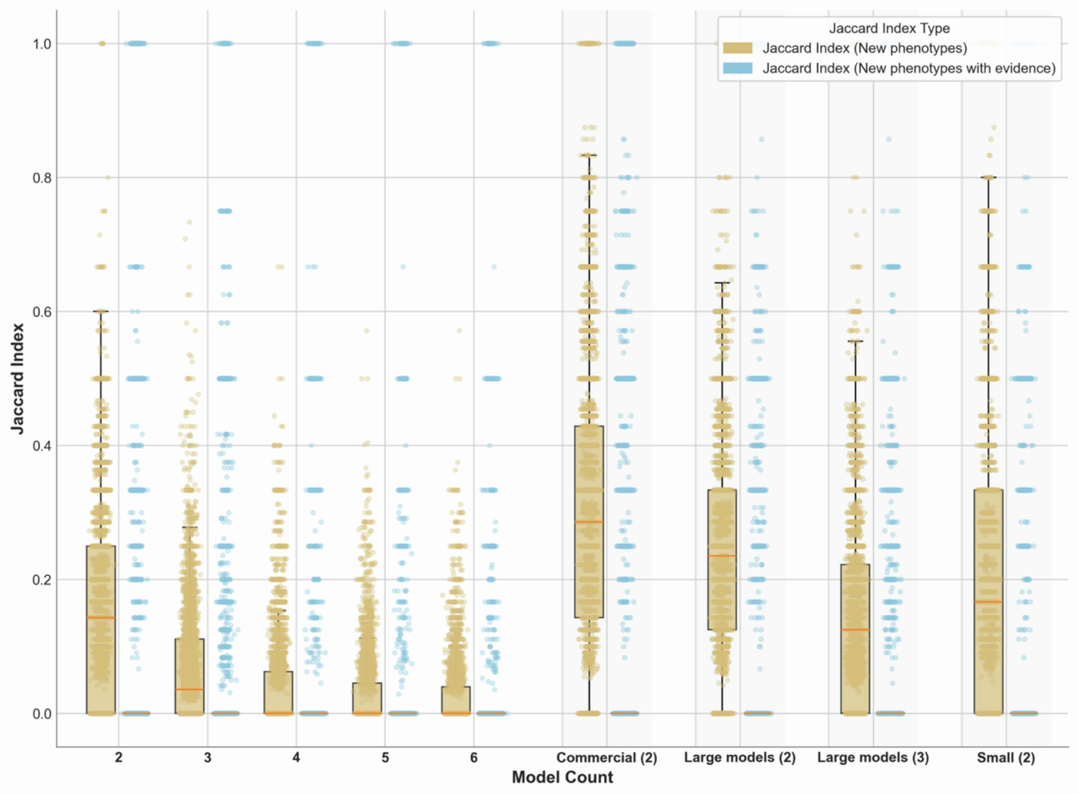

Fig. S4. Overlap analysis and comparison against the Orphanet dataset with a focus on externalized "novel" phenotypes, when considering incrementally larger subsets of LLMs. The results are almost identical to the ones in Fig. S3. A major difference can be noted in the increased overlap on novel phenotypes with evidence.

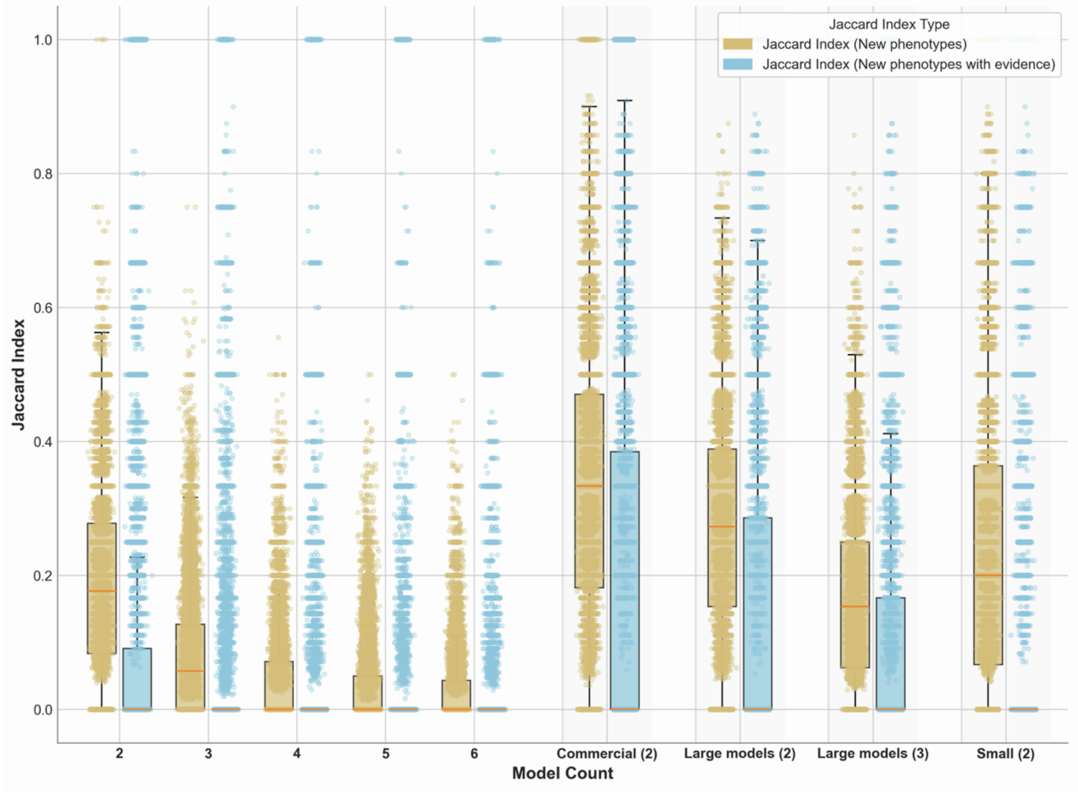

Fig. S5. Overlap analysis and comparison against the HPOA dataset with a focus on externalized "novel" genes, when considering incrementally larger subsets of LLMs. Although the Jaccard index is generally very low, Commercial models display a high degree of overlap both overall as well as on new genes with evidence.

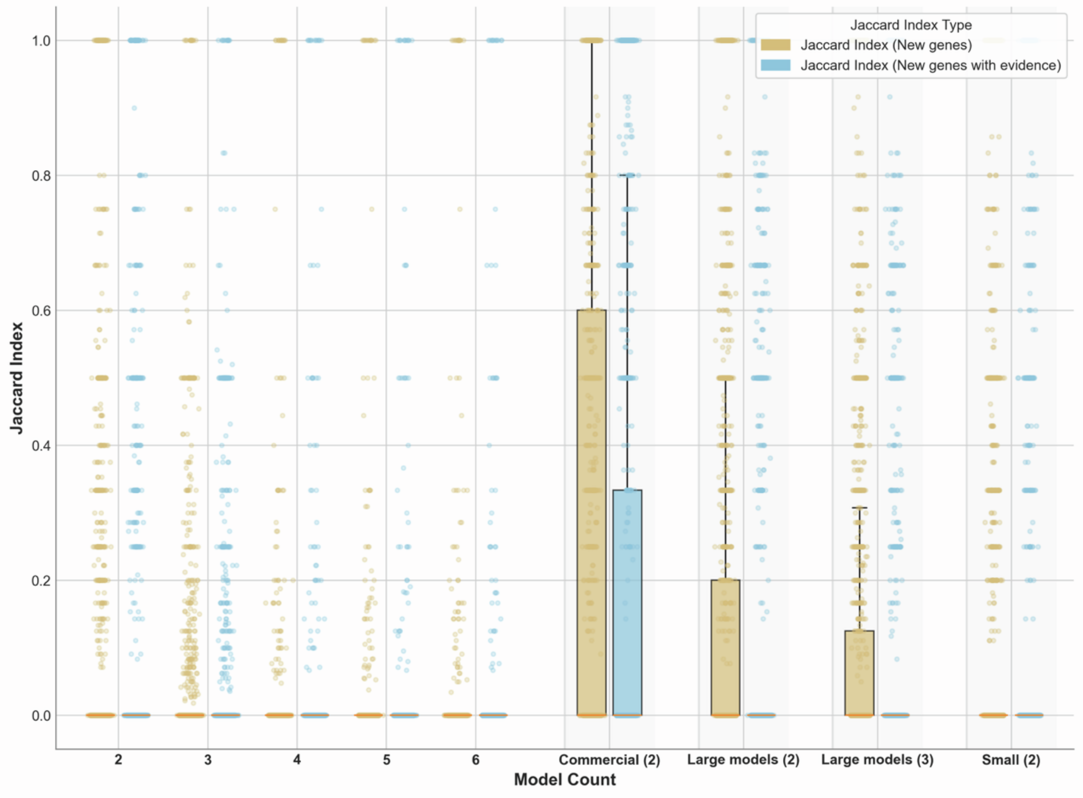

Fig. S6. Overlap analysis and comparison against the Orphanet dataset with a focus on externalized "novel" genes when considering incrementally larger subsets of LLMs. A major difference is the decreased overlap on new genes with evidence of the Commercial models, which is most likely due to the increased number of conditions underpinning the dataset – and hence the higher chance of divergence.

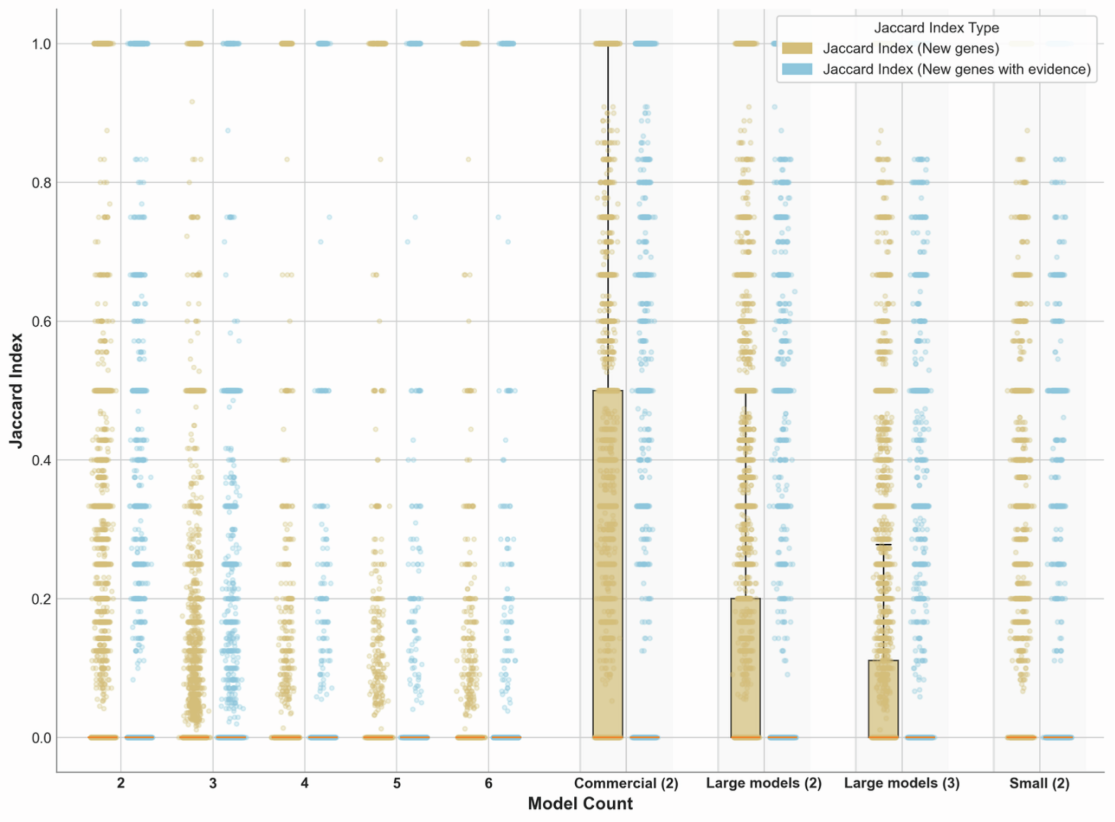

Fig. S7. Coverage and correlation of the LIRICAL ranking results when considering the model-specific knowledge base – i.e., the disease-phenotype associations produced by each model individually (see Table S2 for concrete counts). The coverage shows how many of the diseases comprised in the Phenopackets gold standard corpus are covered by the actual model, compared to the coverage of the various evaluation strategies (LIRICAL, direct prompting with exact or broader matching). LIRICAL and direct prompting with exact matching follow equivalent comparison strategies (i.e., the disease ID must be the same in the gold standard and the test set). Experimental results using LIRICAL, however, provide twice the disease coverage. Unsurprisingly, direct evaluation with broader matching produces an almost complete coverage; most of the conditions comprised in the Phenopackets corpus are sub-types and, hence, ignoring the sub-type via the relaxed matching strategy leads to an increased coverage.

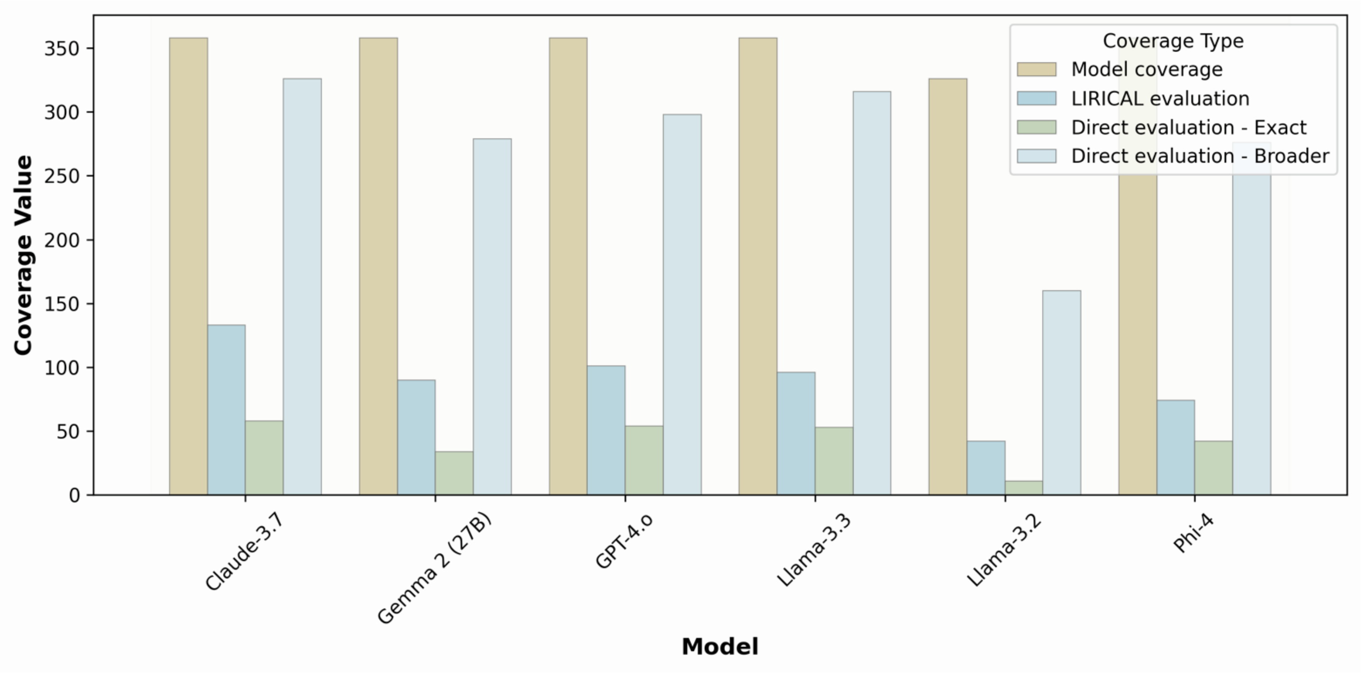

Fig. S8. Coverage and correlation of the LIRICAL ranking results when considering the Common knowledge base – i.e., the disease-phenotype associations for the conditions that are common across all knowledge bases (see Table S2 for concrete counts). The results are in principle a mirror of the results reported for the Model-specific knowledge bases in Fig. S7.

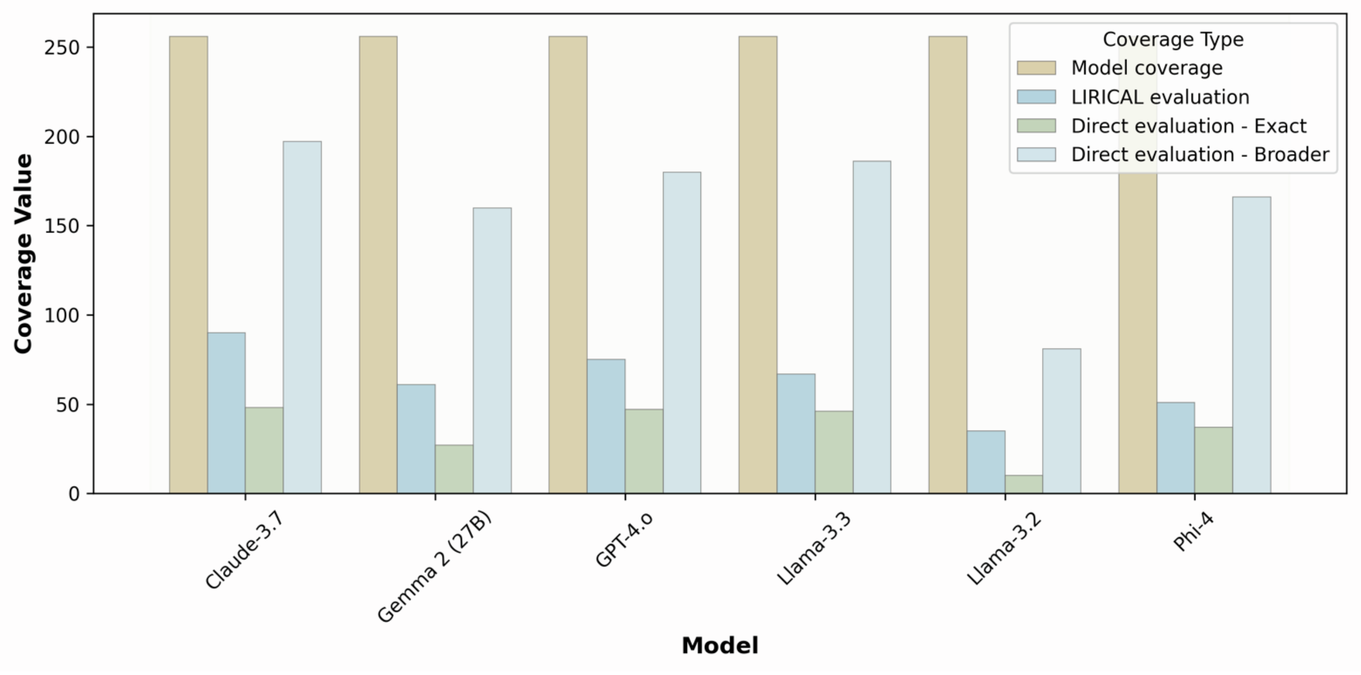

Fig. S9. Overlap analysis using Jaccard index between the disease-phenotype associations underpinning the Model-specific LIRICAL ranking evaluation and the HPOA / Orphanet datasets. Median values across all models follow the same pattern as the general overlap analysis. The subset of phenotype models considered in this context are, hence, not closer in terms of overlap to the reference knowledge bases.

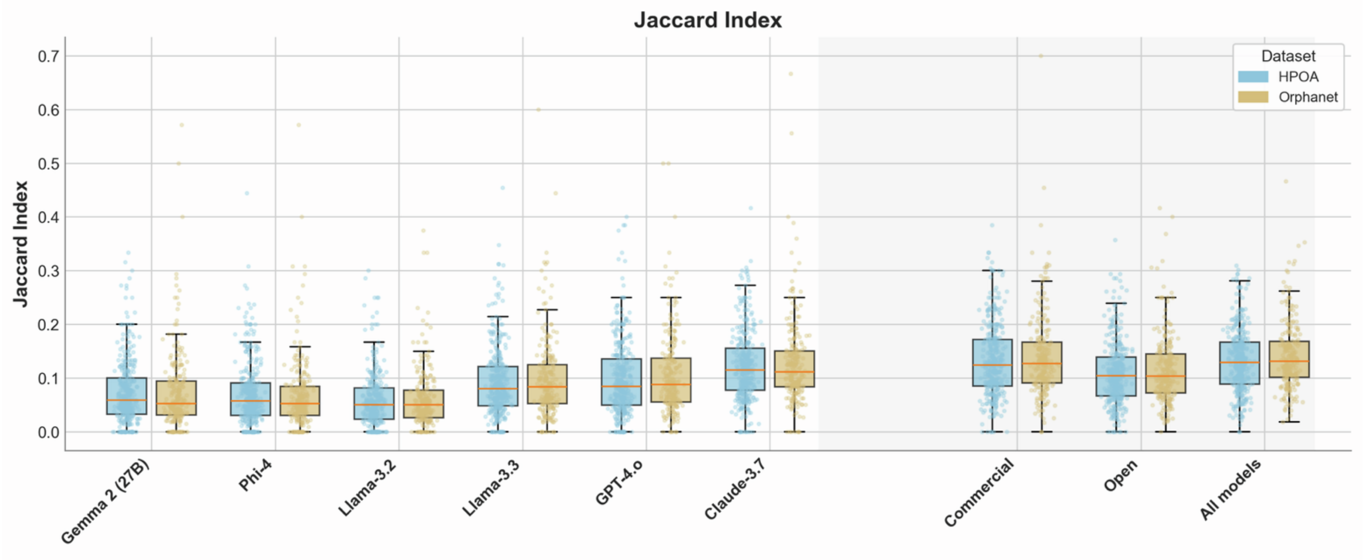

Fig. S10. Overlap analysis using normalized semantic similarity between the disease-phenotype associations underpinning the Model-specific LIRICAL ranking evaluation and the HPOA / Orphanet datasets. Similar to Fig. S9, median values across all models follow the same pattern as the general analysis.

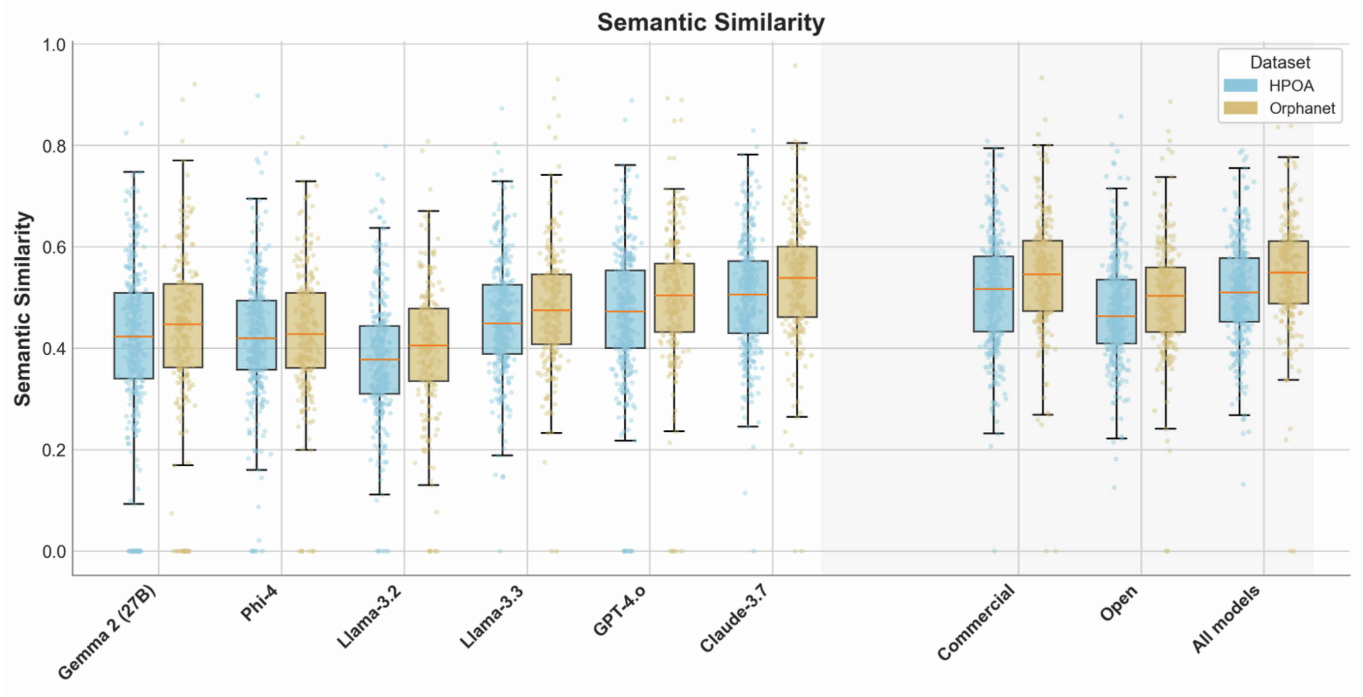

Fig. S11. Overlap analysis using Jaccard index between the disease-phenotype associations underpinning the Common LIRICAL ranking evaluation and the HPOA / Orphanet datasets. The Common dataset consisted of conditions that were covered by all Model-specific knowledge bases. The median of the Jaccard index values is again similar to the one presented in Fig. S9, as well as to the general dataset analysis depicted in Fig. 1.

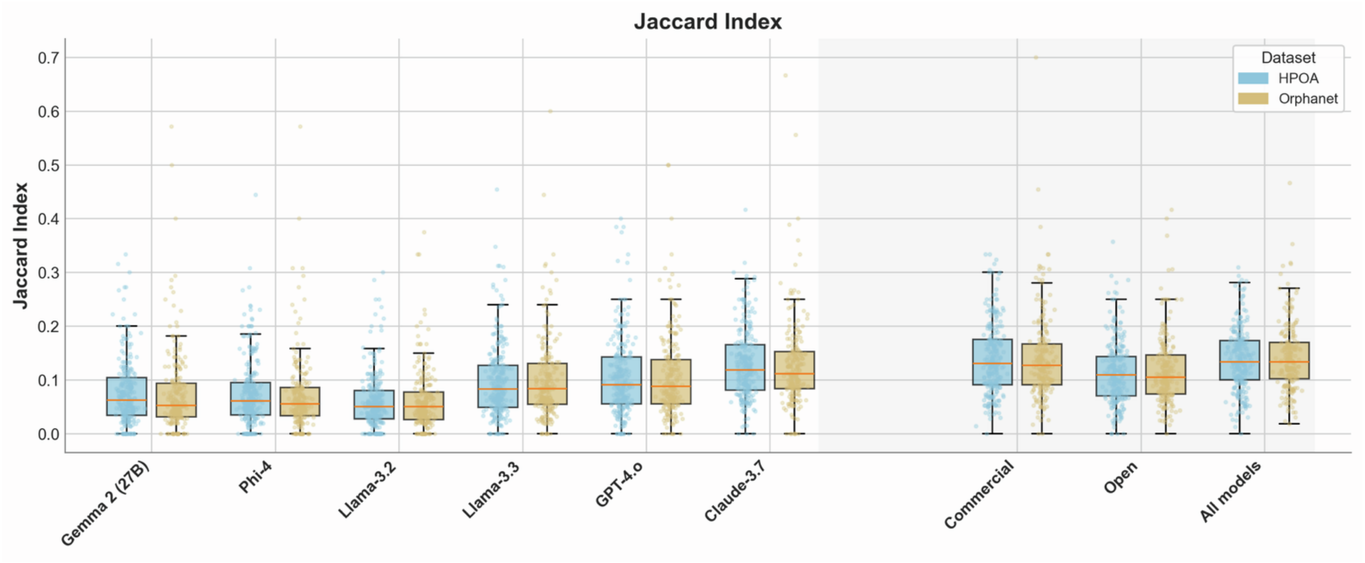

Fig. S12. Overlap analysis using normalized semantic similarity between the disease-phenotype associations underpinning the Common LIRICAL ranking evaluation and the HPOA / Orphanet datasets, with the median value mirroring the values in Fig. 1 and S10.

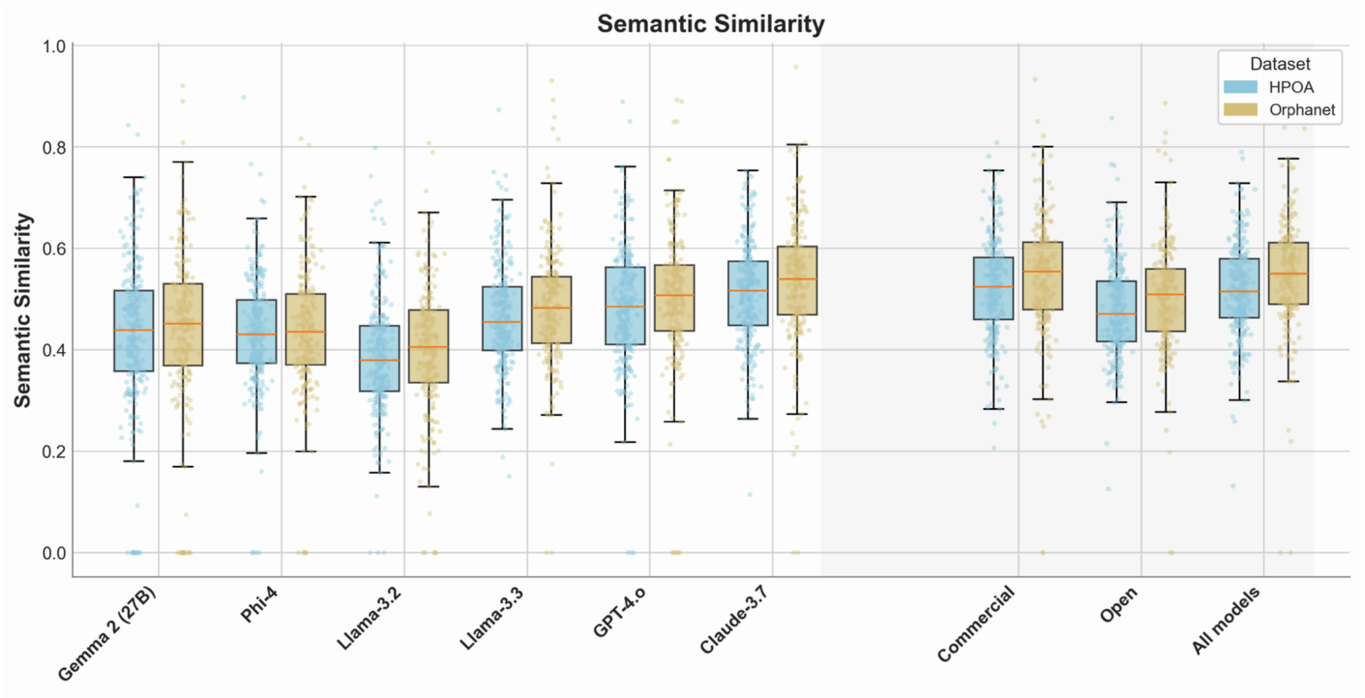

Fig. S13. Distributions of pairwise Jaccard index and semantic similarity between the phenotype sets produced by various LLMs and reference knowledge base Orphanet, group by the high-level type of diseases (according to Orphanet): Genetic, Neoplastic and Other (i.e., neither Genetic nor Neoplastic). The results are comparable to Fig. 2A applied to Orphanet.

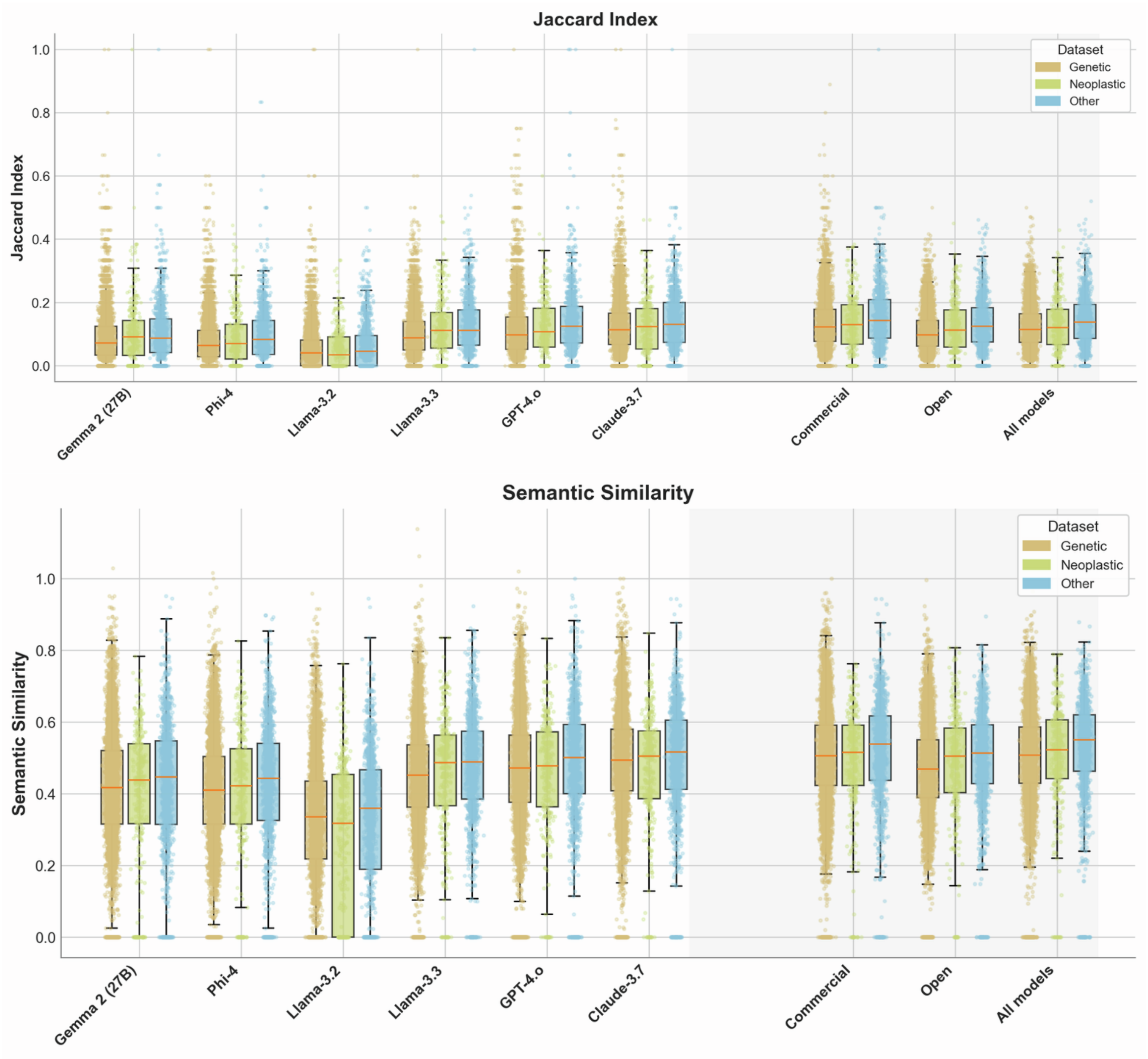

Fig. S14. Distributions of pairwise Jaccard index and semantic similarity between the phenotype sets produced by various LLMs and reference knowledge base Orphanet, group by the prevalence defined in Orphanet. The results are comparable to Fig. 2A applied to Orphanet.

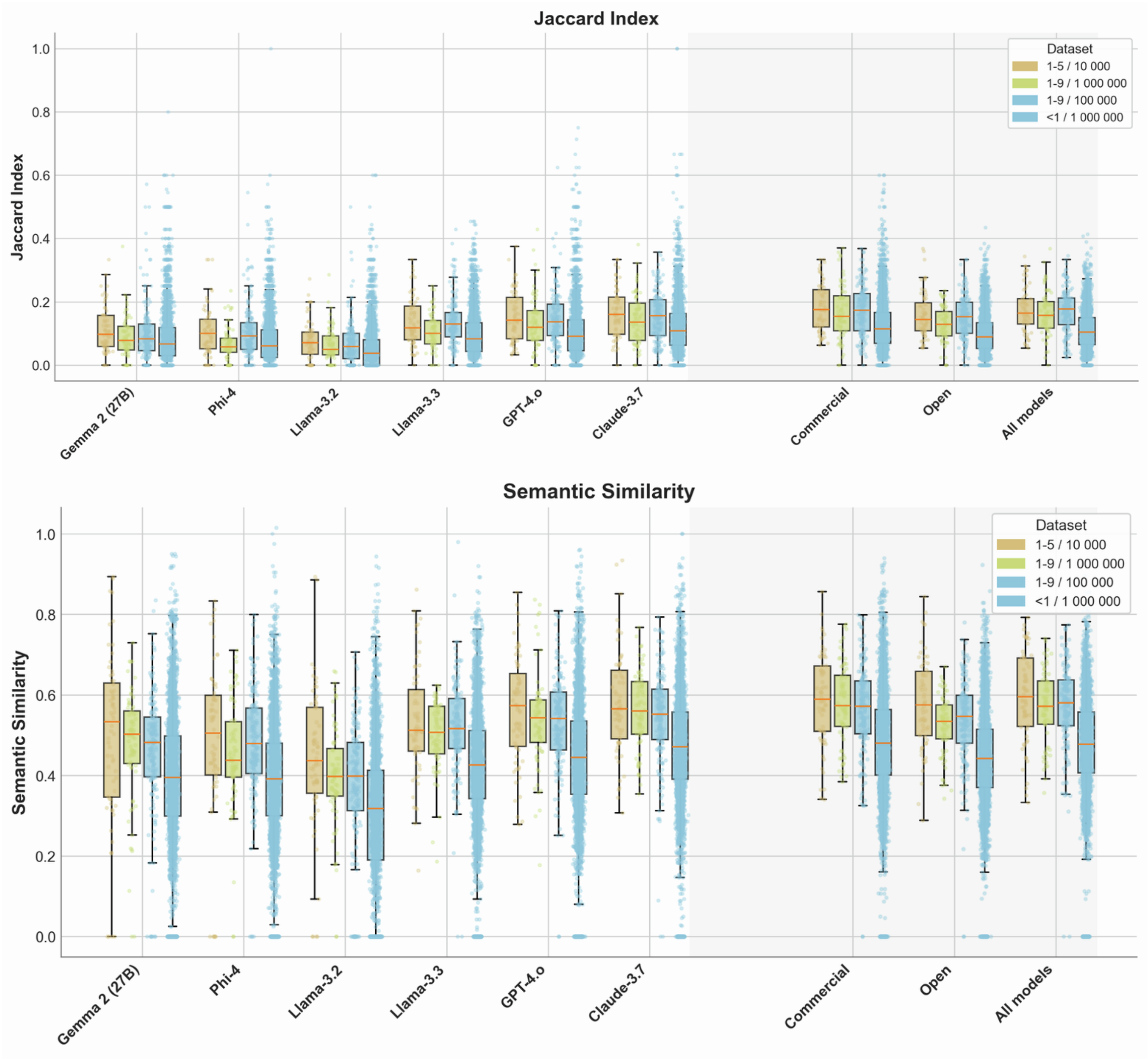

Fig. S15. Distribution of new classes of phenotypes produced by the LLMs. A new class of phenotype denotes phenotypes belonging to top-level HPO abnormalities (e.g., Digestive, Respiratory, Growth) previously not covered by the base Orphanet model.

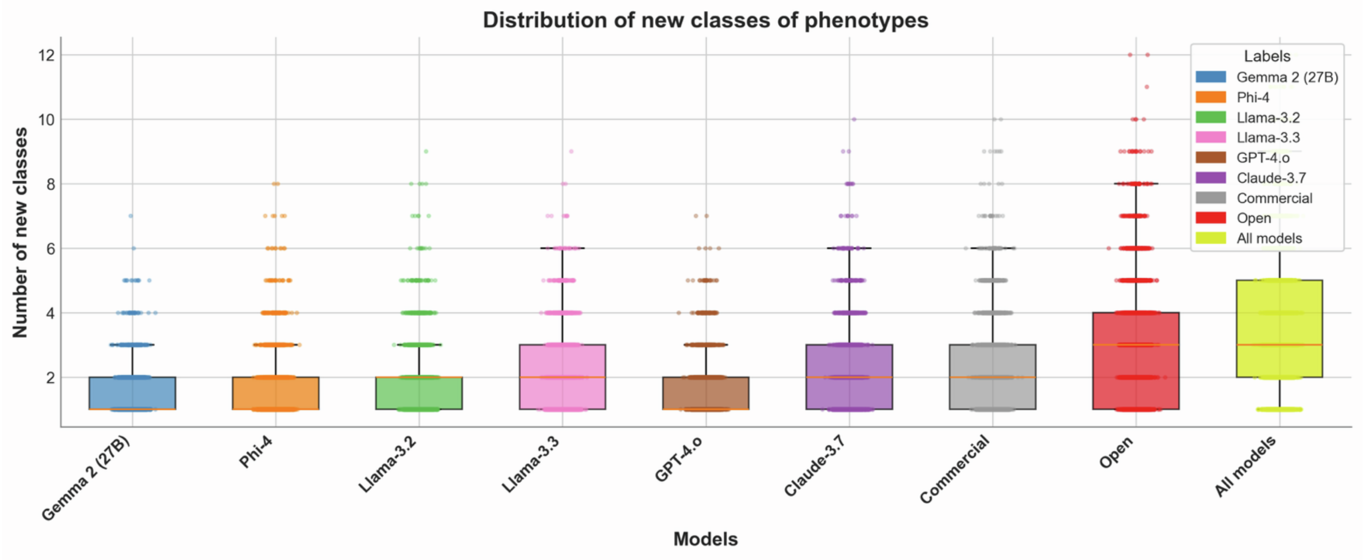

Fig. S16. Distribution of the percentages of phenotype entries produced by the LLMs that were matched or unable to be matched to HPO terms. There is a marked difference between the two distributions with a median value of 0 for entries that were not matched and an average of 120% for entries that were matched to HPO terms across all LLMs. The percentages of matched entries can reach values over 100% because of the multi-phenotype nature of some of the items produced by the LLMs. For example, "*Pierre Robin sequence (micrognathia, glossoptosis, cleft palate)*" as a single entry was decomposed and mapped to four individual HPO terms: Pierre Robin sequence, micrognathia, glossoptosis, cleft palate. This is in line with the standard annotation process employed by Orphanet and the Human Phenotype Ontology teams and can be observed in the existing knowledge bases.

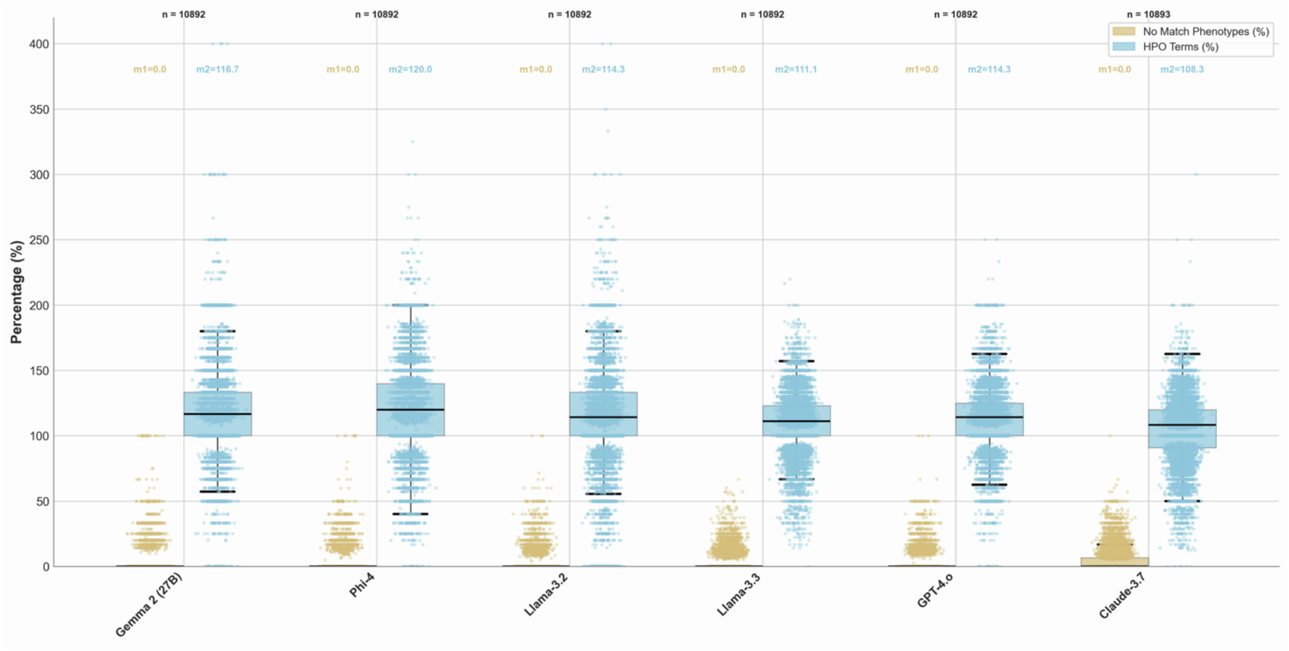

Table S1. Distributions of pairwise Jaccard index and semantic similarity between the phenotype sets produced by various LLMs and reference knowledge bases HPOA (HPO annotations), Orphanet and Orphanet (freq) – the latter denoting subset of Orphanet retaining only the frequent, very frequent and obligate phenotypes.

	**Jaccard Index**					**Semantic Similarity**				
	Med	Q1	Q3	1.5Q1	1.5Q3	Med	Q1	Q3	1.5Q1	1.5Q3
	**HPOA**									
**Claude-3.7**	0.105	0.058	0.161	0.087	0.241	0.467	0.37	0.557	0.555	0.836
**GPT-4.o**	0.094	0.048	0.15	0.072	0.225	0.447	0.338	0.544	0.507	0.816
**Llama-3.3 (70B)**	0.086	0.045	0.143	0.068	0.214	0.432	0.336	0.526	0.504	0.789
**Gemma 2 (27B)**	0.068	0.019	0.125	0.028	0.188	0.395	0.277	0.5	0.416	0.75
**Phi-4 (14B)**	0.067	0.022	0.111	0.033	0.167	0.395	0.298	0.491	0.447	0.736
**Llama-3.2 (3B)**	0.037	0	0.083	0	0.124	0.32	0.173	0.425	0.259	0.637
**Commercial**	0.115	0.067	0.167	0.101	0.251	0.483	0.389	0.568	0.584	0.852
**Open**	0.093	0.054	0.138	0.081	0.207	0.445	0.364	0.527	0.546	0.79
**All models**	0.105	0.065	0.151	0.098	0.226	0.478	0.393	0.557	0.59	0.836
	**Orphanet**									
**Claude-3.7**	0.111	0.068	0.167	0.102	0.251	0.502	0.415	0.583	0.622	0.874
**GPT-4.o**	0.094	0.053	0.148	0.08	0.222	0.48	0.389	0.568	0.584	0.852
**Llama-3.3 (70B)**	0.089	0.053	0.138	0.08	0.207	0.465	0.377	0.545	0.566	0.818
**Gemma 2 (27B)**	0.067	0.033	0.111	0.05	0.167	0.427	0.324	0.525	0.486	0.788
**Phi-4 (14B)**	0.062	0.029	0.105	0.044	0.158	0.42	0.325	0.511	0.488	0.766
**Llama-3.2 (3B)**	0.038	0	0.077	0	0.115	0.348	0.226	0.444	0.339	0.666
**Commercial**	0.125	0.08	0.179	0.12	0.268	0.515	0.432	0.596	0.648	0.894
**Open**	0.103	0.065	0.15	0.098	0.225	0.48	0.403	0.559	0.605	0.839
**All models**	0.123	0.08	0.172	0.12	0.258	0.521	0.44	0.594	0.66	0.891
	**Orphanet (freq)**									
**Claude-3.7**	0.121	0.067	0.182	0.101	0.273	0.492	0.4	0.584	0.6	0.876
**GPT-4.o**	0.111	0.056	0.169	0.084	0.254	0.471	0.366	0.569	0.549	0.853
**Llama-3.3 (70B)**	0.095	0.053	0.154	0.08	0.231	0.452	0.352	0.541	0.528	0.812
**Gemma 2 (27B)**	0.083	0.036	0.143	0.054	0.214	0.415	0.304	0.527	0.456	0.79
**Phi-4 (14B)**	0.074	0.029	0.13	0.044	0.195	0.409	0.305	0.51	0.458	0.765
**Llama-3.2 (3B)**	0.043	0	0.091	0	0.137	0.328	0.194	0.439	0.291	0.658
**Commercial**	0.128	0.077	0.188	0.115	0.282	0.506	0.417	0.595	0.625	0.892
**Open**	0.1	0.059	0.15	0.088	0.225	0.47	0.382	0.557	0.573	0.836
**All models**	0.113	0.069	0.167	0.104	0.251	0.51	0.425	0.592	0.637	0.888

Table S2. List of diseases with high phenotypic semantic similarity across models.

Model	Disease name	Normalized semantic similarity	Number of phenotypes in the base model
**Claude-3.7**	Li-Fraumeni syndrome	0.9603	35
	Familial short QT syndrome	0.9576	9
	Partial chromosome Y deletion	0.9241	6
	Bleeding disorder in hemophilia A carriers	0.9035	12
	Spastic ataxia with congenital miosis	0.8976	8
	Myoclonus-dystonia syndrome	0.8787	11
**GPT-4.o**	Li-Fraumeni syndrome	0.9734	35
	Microcornea-posterior megalolenticonus-persistent fetal vasculature-coloboma syndrome	0.9199	7
	Episodic ataxia type 3	0.9187	7
	Primary hyperoxaluria type 2	0.89292	6
	Catecholaminergic polymorphic ventricular tachycardia	0.8893	10
	Hinman syndrome	0.8824	7
**Llama-3.3 (70B)**	Isolated focal cortical dysplasia	0.9812	21
	Li-Fraumeni syndrome	0.9404	35
	Familial short QT syndrome	0.93063	9
	Idiopathic hypersomnia	0.9226	11
	Brugada syndrome	0.8933	12
	Hypoxanthine guanine phosphoribosyltransferase partial deficiency	0.8723	8
	Knuckle pads-leukonychia-sensorineural deafness-palmoplantar hyperkeratosis syndrome	0.8577	7
**Gemma 2 (27B)**	Li-Fraumeni syndrome	0.9957	35
	Autosomal dominant focal dystonia, DYT25 type	0.9504	7
	Microcornea-posterior megalolenticonus-persistent fetal vasculature-coloboma syndrome	0.9199	7
	Catecholaminergic polymorphic ventricular tachycardia	0.8901	10
	Partial chromosome Y deletion	0.8890	6
	Pectus excavatum-macrocephaly-dysplastic nails syndrome	0.8639	15
	Knuckle pads-leukonychia-sensorineural deafness-palmoplantar hyperkeratosis syndrome	0.8577	7
**Phi-4 (14B)**	Primary dystonia, DYT21 type	0.9648	9
	Li-Fraumeni syndrome	0.9579	35
	Episodic ataxia type 3	0.8946	7
	Knuckle pads-leukonychia-sensorineural deafness-palmoplantar hyperkeratosis syndrome	0.8577	7
	Microcornea-posterior megalolenticonus-persistent fetal vasculature-coloboma syndrome	0.8538	7
**Llama-3.2 (7B)**	Li-Fraumeni syndrome	0.9579	35
	Microcornea-posterior megalolenticonus-persistent fetal vasculature-coloboma syndrome	0.9199	7
	Spondyloepimetaphyseal dysplasia, Handigodu type	0.9148	29
	Partial chromosome Y deletion	0.8856	6
	Knuckle pads-leukonychia-sensorineural deafness-palmoplantar hyperkeratosis syndrome	0.8514	7
	Turcot syndrome with polyposis	0.8293	42
	IMAGe syndrome	0.8176	13

Table S3. Distributions of the percentage of new phenotypes and new phenotypes with scientific evidence and new genes and new genes with scientific evidence across diseases already associated with phenotype/genes and diseases without annotations

**Genes**										
	**Med**	**Q1**	**Q3**	**1.5Q1**	**1.5Q3**	**Med**	**Q1**	**Q3**	**1.5Q1**	**1.5Q3**
Diseases with existing associations										
	**New entries**					**New entries with evidence**				
**Claude-3.7**	0.75	0.5	1	0.75	1	0	0	0.25	0	0.375
**GPT-4.o**	1	0.5	1	0.75	1	0	0	0.25	0	0.375
**Llama-3.3 (70B)**	1	0.5	1	0.75	1	0	0	0	0	0
**Gemma 2 (27B)**	1	1	1	1	1	0	0	0	0	0
**Phi-4 (14B)**	1	1	1	1	1	0	0	0	0	0
**Llama-3.2 (3B)**	1	1	1	1	1	0	0	0	0	0
Diseases without existing associations										
**Claude-3.7**						0	0	0.5	0	0.75
**GPT-4.o**						0	0	1	0	1
**Llama-3.3 (70B)**						0	0	0.33	0	0.495
**Gemma 2 (27B)**						0	0	0.5	0	0.75
**Phi-4 (14B)**						0	0	0	0	0
**Llama-3.2 (3B)**						0	0	0	0	0
**Phenotypes**										
Diseases with existing associations										
	**New entries**					**New entries with evidence**				
**Claude-3.7**	0.52	0.38	0.67	0.57	1	0	0	0.4	0	0.6
**GPT-4.o**	0.47	0.33	0.62	0.495	0.93	0	0	0.5	0	0.75
**Llama-3.3 (70B)**	0.56	0.4	0.7	0.6	1	0	0	0.38	0	0.57
**Gemma 2 (27B)**	0.5	0.33	0.67	0.495	1	0	0	0.5	0	0.75
**Phi-4 (14B)**	0.57	0.4	0.71	0.6	1	0	0	0.33	0	0.495
**Llama-3.2 (3B)**	0.67	0.5	0.8	0.75	1	0	0	0.25	0	0.375
Diseases without existing associations										
**Claude-3.7**						0	0	0.25	0	0.375
**GPT-4.o**						0	0	0.33	0	0.495
**Llama-3.3 (70B)**						0	0	0.23	0	0.345
**Gemma 2 (27B)**						0	0	0.25	0	0.375
**Phi-4 (14B)**						0	0	0.25	0	0.375
**Llama-3.2 (3B)**						0	0	0.18	0	0.27

Table S4. Coverage of diseases present in the Phenopackets corpus across the various knowledge bases created from the phenotype sets externalized by the models. The last three entries (*Commercial*, *Open* and *All*) represent combo models where the phenotype set was created via a set union of the phenotype sets of the underpinning models. *Commercial* includes Claude and GPT, *Open* the rest of the models and *All* – all models.

Knowledge base	Number of diseases	Number of patients
**HPOA**	514	8151
**ORPHANET**	279	5474
**COMMON**	256	5122
**Claude-3.7**	358	6764
**GPT-4.o**	358	6764
**Llama-3.3 (70B)**	358	6764
**Gemma 2 (27B)**	358	6764
**Phi-4 (14B)**	358	6764
**Llama-3.2 (3B)**	326	6340
**Commercial**	358	6764
**Open**	358	6764
**All models**	358	6764

Table S5. Top ranked diseases using direct prompting for differential diagnosis across models.

**Exact matching of the disease codes and names**		
Model	**Disease name**	**Number of patients**
**Claude-3.7**	Neurofibromatosis, type 1	231
	Holt-Oram syndrome	156
	Greig cephalopolysyndactyly syndrome	51
	Marfan syndrome	50
	Gillespie syndrome	35
	KBG syndrome	32
	Pallister-Hall syndrome	21
	DiGeorge syndrome	21
	Pseudopseudohypoparathyroidism	15
	Metachondromatosis	12
**GPT-4.o**	Neurofibromatosis, type 1	339
	Holt-Oram syndrome	149
	Greig cephalopolysyndactyly syndrome	51
	Marfan syndrome	51
	Aarskog-Scott syndrome	45
	Gillespie syndrome	28
	Pallister-Hall syndrome	21
	Acromicric dysplasia	13
	Sulfite oxidase deficiency	12
	Cleidocranial dysplasia	8
**Llama-3.3 (70B)**	Neurofibromatosis, type 1	331
	Holt-Oram syndrome	156
	Marfan syndrome	51
	Greig cephalopolysyndactyly syndrome	50
	Aarskog-Scott syndrome	48
	Gillespie syndrome	26
	DiGeorge syndrome	22
	Pallister-Hall syndrome	19
	Sulfite oxidase deficiency	16
	Desmosterolosis	10
**Gemma 2 (27B)**	Neurofibromatosis, type 1	384

	Holt-Oram syndrome	156
	Marfan syndrome	50
	Greig cephalopolysyndactyly syndrome	42
	Ehlers-Danlos syndrome, vascular type	31
	Pseudohypoparathyroidism Ia	24
	DiGeorge syndrome	20
	Lipodystrophy, familial partial, type 2	15
	Gillespie syndrome	10
	Aarskog-Scott syndrome	8
**Phi-4 (14B)**	Neurofibromatosis, type 1	227
	Holt-Oram syndrome	123
	Marfan syndrome	47
	Greig cephalopolysyndactyly syndrome	44
	Pseudohypoparathyroidism Ia	25
	Gillespie syndrome	16
	DiGeorge syndrome	12
	Pallister-Hall syndrome	11
	Lipodystrophy, familial partial, type 2	11
	Adrenal hyperplasia, congenital, due to 21-hydroxylase deficiency	10
**Llama-3.2 (3B)**	Neurofibromatosis, type 1	153
	Marfan syndrome	25
	Pseudohypoparathyroidism Ia	11
	DiGeorge syndrome	7
	Adrenal hyperplasia, congenital, due to 21-hydroxylase deficiency	5
	Holt-Oram syndrome	4
	Greig cephalopolysyndactyly syndrome	2
	Ehlers-Danlos syndrome, vascular type	2
	Peutz-Jeghers syndrome	1
	Jervell and Lange-Nielsen syndrome	1
	Apert syndrome	1
Broader matching of the disease codes and names		
**Claude-3.7**	Developmental and epileptic encephalopathy 4	292
	Neurofibromatosis, type 1	246
	Developmental and epileptic encephalopathy 11	160
	Holt-Oram syndrome	156
	Mitochondrial DNA depletion syndrome 13 (encephalomyopathic type)	88
	Spinocerebellar ataxia 29, congenital nonprogressive	82
	Pseudohypoparathyroidism Ia	81
	Leber congenital amaurosis 6	79
	Loeys-Dietz syndrome 5	75
	Adrenal hyperplasia, congenital, due to 21-hydroxylase deficiency	64
**GPT-4.o**	Neurofibromatosis, type 1	344
	Holt-Oram syndrome	156
	Developmental and epileptic encephalopathy 4	139
	Developmental and epileptic encephalopathy 11	94
	OMIM:615471 Mitochondrial DNA depletion syndrome 13 (encephalomyopathic type)	78
	Loeys-Dietz syndrome 5	75
	Leber congenital amaurosis 6	75
	Spinocerebellar ataxia 29, congenital nonprogressive	75
	Kabuki syndrome 2	72
	Lipodystrophy, familial partial, type 2	71

**Llama-3.3 (70B)**	Neurofibromatosis, type 1	358
	Developmental and epileptic encephalopathy 4	222
	Holt-Oram syndrome	156
	Developmental and epileptic encephalopathy 11	147
	Leber congenital amaurosis 6	77
	Loeys-Dietz syndrome 5	75
	Mitochondrial DNA depletion syndrome 13 (encephalomyopathic type)	71
	Intellectual developmental disorder with autism and macrocephaly	68
	Pseudohypoparathyroidism Ia	67
	Kabuki syndrome 2	63
**Gemma 2 (27B)**	Neurofibromatosis, type 1	386
	Holt-Oram syndrome	156
	Developmental and epileptic encephalopathy 4	123
	Mitochondrial DNA depletion syndrome 13 (encephalomyopathic type)	81
	Leber congenital amaurosis 6	77
	Loeys-Dietz syndrome 5	75
	Developmental and epileptic encephalopathy 11	73
	Kabuki syndrome 2	63
	Pseudohypoparathyroidism Ia	61
	Adrenal hyperplasia, congenital, due to 21-hydroxylase deficiency	54
**Phi-4 (14B)**	Neurofibromatosis, type 1	317
	Developmental and epileptic encephalopathy 4	167
	Holt-Oram syndrome	136
	Developmental and epileptic encephalopathy 11	116
	Loeys-Dietz syndrome 5	72
	Leber congenital amaurosis 6	66
	Pseudohypoparathyroidism Ia	62
	Mitochondrial DNA depletion syndrome 13 (encephalomyopathic type)	61
	Loeys-Dietz syndrome 2	52
	Adrenal hyperplasia, congenital, due to 21-hydroxylase deficiency	51
**Llama-3.2 (3B)**	Neurofibromatosis, type 1	295
	Lipodystrophy, familial partial, type 2	106
	Adrenal hyperplasia, congenital, due to 21-hydroxylase deficiency	44
	Pseudohypoparathyroidism Ia	42
	Marfan syndrome	41
	Developmental and epileptic encephalopathy 4	33
	Leber congenital amaurosis 6	32
	Developmental and epileptic encephalopathy 11	20
	Ehlers-Danlos syndrome, vascular type	20
	Mitochondrial DNA depletion syndrome 13 (encephalomyopathic type)	19

Table S6. Top 10 most similar disease profiles produced by the commercial models when compared to the Orphanet profile grouped by the disease classification according to Orphanet. The table lists the normalized similarity metric and the size of the Orphanet profile in terms of HPO terms

Disease name	Normalized semantic similarity	Size of disease profile in Orphanet
**Endocrine**		
Pendred syndrome	0.8231	15
Temple syndrome due to paternal 14q32.2 hypomethylation	0.8065	28
Pseudopseudohypoparathyroidism	0.7966	18
Palmoplantar keratoderma-XX sex reversal-predisposition to squamous cell carcinoma syndrome	0.7946	3
Autoimmune polyendocrinopathy type 2	0.7794	12
46,XY difference of sex development due to 5-alpha-reductase 2 deficiency	0.7763	10
Temple syndrome due to maternal uniparental disomy of chromosome 14	0.7737	35
Pseudohypoparathyroidism type 2	0.7725	16
Colobomatous microphthalmia-obesity-hypogenitalism-intellectual disability syndrome	0.7664	14
Brain-lung-thyroid syndrome	0.7643	60
**Developmental anomalies during embryogenesis**		
Deafness-infertility syndrome	1.0	3
Partial chromosome Y deletion	0.9344	6
Gingival fibromatosis-progressive deafness syndrome	0.9162	4
MYH9-related disease	0.8942	16
Camptodactyly-taurinuria syndrome	0.8673	4
Waardenburg syndrome type 2	0.8601	12
Isolated focal cortical dysplasia	0.85	21
Spina bifida-hypospadias syndrome	0.8497	3
Corneal dystrophy-perceptive deafness syndrome	0.8482	5
Hereditary neurocutaneous malformation	0.8464	4
**Neurological**		
ABeta amyloidosis, Dutch type	0.9401	10
Spastic ataxia with congenital miosis	0.9197	8
Episodic ataxia type 3	0.8979	7
Myoclonus-dystonia syndrome	0.8787	11
COASY protein-associated neurodegeneration	0.8605	13
Isolated focal cortical dysplasia	0.85	21
Spina bifida-hypospadias syndrome	0.8497	3
Hereditary neurocutaneous malformation	0.8464	4
Primary dystonia, DYT13 type	0.8406	14
Autosomal dominant spastic paraplegia type 13	0.8398	16
**Urogenital**		
Spina bifida-hypospadias syndrome	0.8497	3
Bladder exstrophy	0.7969	13
Palmoplantar keratoderma-XX sex reversal-predisposition to squamous cell carcinoma syndrome	0.7946	3
46,XY difference of sex development due to 5-alpha-reductase 2 deficiency	0.7763	10
Leydig cell hypoplasia	0.7286	23
46,XX ovotesticular difference of sex development	0.7239	14
46,XY partial gonadal dysgenesis	0.7087	43
Exstrophy-epispadias complex	0.704	40
Mayer-Rokitansky-Küster-Hauser syndrome	0.696	20
Aarskog-Scott syndrome	0.6908	49
**Skin**		
Waardenburg syndrome type 2	0.8607	12
Localized dystrophic epidermolysis bullosa, pretibial form	0.8299	35
Pseudopseudohypoparathyroidism	0.7966	18

Palmoplantar keratoderma-XX sex reversal-predisposition to squamous cell carcinoma syndrome	0.7946	3
Pachydermoperiostosis	0.7892	41
Trichorhinophalangeal syndrome type 2	0.7659	38
Waardenburg syndrome type 1	0.7651	32
Oculocutaneous albinism type 2	0.7634	24
Tuberous sclerosis complex	0.7621	62
Pili torti-developmental delay-neurological abnormalities syndrome	0.762	10
**Immunological**		
WHIM syndrome	0.8532	34
Griscelli syndrome type 2	0.7582	19
Selective IgM deficiency	0.7396	64
X-linked severe congenital neutropenia	0.66931	3
Herpes simplex virus encephalitis	0.6917	30
Familial hemophagocytic lymphohistiocytosis	0.6916	45
Recurrent infections associated with rare immunoglobulin isotypes deficiency	0.6909	47
X-linked lymphoproliferative disease due to XIAP deficiency	0.6856	22
Chédiak-Higashi syndrome	0.6751	76
Majeed syndrome	0.6705	32
**Ophthalmic**		
Spastic ataxia with congenital miosis	0.9197	8
Corneal dystrophy-perceptive deafness syndrome	0.8482	5
X-linked intellectual disability-seizures-psoriasis syndrome	0.7989	5
Pseudopseudohypoparathyroidism	0.7966	18
Galactosialidosis	0.7934	9
Eales disease	0.7924	30
Nance-Horan syndrome	0.792	19
Persistent placoid maculopathy	0.7864	8
Microtia-eye coloboma-imperforation of the nasolacrimal duct syndrome	0.7782	4
Congenital hereditary endothelial dystrophy type II	0.775	9
**Gynaecological and obstetric**		
Palmoplantar keratoderma-XX sex reversal-predisposition to squamous cell carcinoma syndrome	0.7946	3
Autoimmune polyendocrinopathy type 2	0.7794	12
46,XY difference of sex development due to 5-alpha-reductase 2 deficiency	0.7763	10
Ataxia-hypogonadism-choroidal dystrophy syndrome	0.738	3
Hereditary breast and/or ovarian cancer syndrome	0.7343	7
46,XX ovotesticular difference of sex development	0.7239	14
46,XY partial gonadal dysgenesis	0.7087	43
Osteosclerosis-ichthyosis-premature ovarian failure syndrome	0.6971	4
Mayer-Rokitansky-Küster-Hauser syndrome	0.696	20
Ovarian hyperstimulation syndrome	0.6846	17
**Otorhinolaryngological**		
Deafness-infertility syndrome	1.0	3
Gingival fibromatosis-progressive deafness syndrome	0.9162	4
MYH9-related disease	0.8942	16
Waardenburg syndrome type 2	0.8607	12
Corneal dystrophy-perceptive deafness syndrome	0.8482	5
Pendred syndrome	0.8231	15
Bartter syndrome type 4	0.7965	36
TARP syndrome	0.7828	55
Waardenburg syndrome type 1	0.7651	32
Waardenburg syndrome	0.757	32
**Inborn errors of metabolism**		
Iminoglycinuria	0.9371	6
Galactosialidosis	0.7934	9
Ethylmalonic encephalopathy	0.7886	18

Glucose-galactose malabsorption	0.7815	15
Primary hyperoxaluria type 3	0.7785	6
Crigler-Najjar syndrome type 1	0.7688	14
Oculocutaneous albinism type 2	0.7634	24
Fanconi-Bickel syndrome	0.76	31
Oculocutaneous albinism type 4	0.7585	15
Oculocutaneous albinism type 1B	0.7554	17
**Systemic and rheumatological**		
ABeta amyloidosis, Dutch type	0.9401	10
ATTRV30M amyloidosis	0.7725	14
Syndromic diarrhea	0.7527	52
Hereditary hemorrhagic telangiectasi	0.7481	36
Marfan syndrome	0.7373	68
Brittle cornea syndrome	0.7316	34
Hereditary angioedema with normal C1Inh	0.721	13
Congenital contractural arachnodactyly	0.7187	21
Hereditary amyloidosis with primary renal involvement	0.7166	43
ABeta amyloidosis, Italian type	0.7128	7
**Bone**		
Pseudopseudohypoparathyroidism	0.7966	18
Galactosialidosis	0.7934	9
Pachydermoperiostosis	0.7892	41
Radio-ulnar synostosis-amegakaryocytic thrombocytopenia syndrome	0.7831	6
Mesomelic dysplasia, Nievergelt type	0.7807	26
Feingold syndrome type 2	0.7719	15
Trichorhinophalangeal syndrome type 2	0.7659	38
ADULT syndrome	0.7554	24
Laurin-Sandrow syndrome	0.7455	28
Orofaciodigital syndrome type 5	0.7425	27
**Renal**		
MYH9-related disease	0.8942	16
Bladder exstrophy	0.7969	13
Pseudopseudohypoparathyroidism	0.7966	18
Bartter syndrome type 4	0.7965	36
Primary hyperoxaluria type 2	0.7785	6
C3 glomerulopathy	0.778	23
Pseudohypoparathyroidism type 2	0.7725	16
Apparent mineralocorticoid excess	0.7622	17
Tuberous sclerosis complex	0.7621	62
Fanconi-Bickel syndrome	0.76	31
**Hepatic**		
Crigler-Najjar syndrome type 1	0.7688	14
Fanconi-Bickel syndrome	0.76	31
Symptomatic form of HFE-related hemochromatosis	0.7541	44
Syndromic diarrhea	0.7527	52
Hereditary hemorrhagic telangiectasia	0.7481	36
HJV or HAMP-related hemochromatosis	0.7298	15
MPI-CDG	0.7121	22
Crigler-Najjar syndrome type 2	0.697	3
Chronic visceral acid sphingomyelinase deficiency	0.6954	47
Neonatal ichthyosis-sclerosing cholangitis syndrome	0.6823	15
**Cardiac**		
Familial atrial fibrillation	0.9105	11
Romano-Ward syndrome	0.8351	13
Familial short QT syndrome	0.8336	9
Atrial septal defect-atrioventricular conduction defects syndrome	0.8143	3
GNB5-related intellectual disability-cardiac arrhythmia syndrome	0.7846	22

ATTRV30M amyloidosis	0.7725	14
Catecholaminergic polymorphic ventricular tachycardia	0.7451	10
Andersen-Tawil syndrome	0.7408	51
Pseudoxanthoma elasticum	0.7331	41
Brugada syndrome	0.7237	12
**Haematological**		
MYH9-related disease	0.8942	16
Bleeding disorder in hemophilia A carriers	0.8805	12
Congenital factor VII deficiency	0.874	12
Beta-thalassemia-X-linked thrombocytopenia syndrome	0.8464	6
Congenital factor II deficiency	0.8439	21
IRIDA syndrome	0.7895	10
Radio-ulnar synostosis-amegakaryocytic thrombocytopenia syndrome	0.7831	6
Bernard-Soulier syndrome	0.7508	21
Congenital factor XI deficiency	0.743	9
Glanzmann thrombasthenia	0.7102	20
**Odontological**		
Gingival fibromatosis-progressive deafness syndrome	0.9162	4
Nance-Horan syndrome	0.792	19
ADULT syndrome	0.7554	24
Gingival fibromatosis-hypertrichosis syndrome	0.7299	13
Bruck syndrome	0.7153	14
Junctional epidermolysis bullosa inversa	0.7058	25
Classical-like Ehlers-Danlos syndrome type 1	0.6673	20
Odonto-onycho dysplasia-alopecia syndrome	0.6604	11
SATB2-associated syndrome due to a pathogenic variant	0.6437	48
Dentinogenesis imperfecta	0.6522	21
**Gastroenterological**		
NTHL1-related attenuated familial adenomatous polyposis	0.8751	15
Turcot syndrome with polyposis	0.837	42
Polymerase proofreading-related adenomatous polyposis	0.803	7
Glucose-galactose malabsorption	0.7815	15
Syndromic diarrhea	0.7527	52
Megacystis-microcolon-intestinal hypoperistalsis syndrome	0.7517	17
Familial adenomatous polyposis	0.7336	40
MPI-CDG	0.7121	22
Waardenburg-Shah syndrome	0.7017	23
Goldberg-Shprintzen megacolon syndrome	0.6908	23
**Infertility**		
Deafness-infertility syndrome	1.0	3
Male infertility due to acephalic spermatozoa	0.9746	7
Partial chromosome Y deletion	0.9344	6
Female infertility due to oocyte meiotic arrest	0.9253	6
Autoimmune polyendocrinopathy type 2	0.7794	12
46,XY difference of sex development due to 5-alpha-reductase 2 deficiency	0.7763	10
Ataxia-hypogonadism-choroidal dystrophy syndrome	0.738	3
Leydig cell hypoplasia	0.7296	23
46,XX ovotesticular difference of sex development	0.7239	14
46,XY partial gonadal dysgenesis	0.7087	43
**Respiratory**		
Lymphangioleiomyomatosis	0.7674	36
Brain-lung-thyroid syndrome	0.7643	60
Hereditary hemorrhagic telangiectasia	0.7481	36
Idiopathic bronchiectasis	0.7255	19
Matthew-Wood syndrome	0.7177	20
Chronic visceral acid sphingomyelinase deficiency	0.6954	47
Primary ciliary dyskinesia	0.692	47

Gaucher disease type 3	0.6901	440
Gaucher disease type 2	0.681	33
Young syndrome	0.6776	5
**Cardiac malformations**		
Atrial septal defect-atrioventricular conduction defects syndrome	0.8143	3
TARP syndrome	0.7828	55
Patent ductus arteriosus-bicuspid aortic valve-hand anomalies syndrome	0.713	8
Familial bicuspid aortic valve	0.7038	12
Noonan syndrome with multiple lentigines	0.6757	50
Holt-Oram syndrome	0.668	29
Truncus arteriosus	0.6542	36
FLNA-related X-linked myxomatous valvular dysplasia	0.6311	24
Cardiac-valvular Ehlers-Danlos syndrome	0.6235	49
Atrial septal defect, sinus venosus type	0.6076	27

LLM knowledge externalization prompt

I'm running experiment to document and quantify the degree of knowledge you as a large language model have. Think and work with me on this.
I'm going to give you the name of a condition (and possible alternate titles) and I want to you respond with everything you know or believe to be relevant about that condition. We will focus on 2 components: phenotypes or clinical features and genes. Respond only to what I've asked.

Some definitions first.
For frequencies, use the following definitions:

* EXCLUDED: Present in 0% of the cases.
* OBLIGATE: Always present, i.e. in 100% of the cases.
* VERY_FREQUENT: Present in 80% to 99% of the cases.
* FREQUENT: Present in 30% to 79% of the cases.
* OCCASIONAL: Present in 5% to 29% of the cases.
* VERY_RARE: Present in 1% to 4% of the cases.

Now the task.

1. Phenotypes - list all phenotypes or clinical features you know are associated with the condition and their frequency in the disease population. If you don't know any phenotypes or clinical features, write 'N/A'. If you don't know the frequency of a phenotype, write 'N/A'.

Return bullet list:
* PHENOTYPE 1 : FREQUENCY
* PHENOTYPE 2 : FREQUENCY
...

2. Genes - list all genes you know are associated with the condition. If you don't know any genes, write 'N/A'.

Return bullet list:
* GENE 1
* GENE 2
...

Make sure you are comprehensive and you include EVERYTHING you know. Return ONLY what I've asked for. Format the results in markdown.

The condition is: %%DISEASE%%

LLM phenotype relevance assessment prompt

You are a medical specialist reader and assessor. You will receive a phenotype, the name of a disease and the abstract of a scientific publication one below each other in the format:

Phenotype: <PHENOTYPE>
Disease: <DISEASE>
Text: <TEXT>

Your task is to read the text, process the text and assess if the phenotype <PHENOTYPE> you have received is associated or a manifestation of the disease <DISEASE> or if the phenotype <PHENOTYPE> is caused by disease <DISEASE>.

Respond with YES or NO, subject to your assessment. Focus on your task, assess if <PHENOTYPE> is caused by <DISEASE> or associated with <DISEASE> based on <TEXT> and respond only with YES or NO. Do not respond with anything else. Respond with YES or NO.

Here are the details:

LLM direct diagnosis prompt

You are a clinical geneticist taking part in a multi-disciplinary meeting that diagnoses rare disease patients.
You will receive below the phenotype profile of the patient as a list.
Your task is to produce a list of max 10 potential diagnoses ranked from the most probable to the least probable.
For each diagnosis in your ranking list the phenotypes that support the diagnosis exactly how they were provided to you.
Do not return any other information. I repeat return strictly only the ranked list and the associated phenotypes supporting each entry - exactly as provided in the input.
Here's the expected format of the output:
```
1. DIAG 1 | Phenotypes :: Phenotype 1; Phenotype 2; Phenotype 3
2. DIAG 2 | Phenotypes :: Phenotype 1; Phenotype 2; Phenotype 3
...
```
Here is the patient information:
